# Inverse analysis of patient-specific parameters of a 3D–0D closed-loop cardiovascular model with an exemplary application to an adult tetralogy of Fallot case

**DOI:** 10.1007/s10237-025-02006-w

**Published:** 2025-09-13

**Authors:** Tahar Arjoune, Christian Bilas, Christian Meierhofer, Heiko Stern, Peter Ewert, Michael W. Gee

**Affiliations:** 1 Mechanics and High Performance Computing Group, Technical University of Munich, Parkring 35, 85748 Garching b. München, Germany; 2 Department of Pediatric Cardiology and Congenital Heart Disease, German Heart Center Munich, Technical University of Munich, Lazarettstraße 36, 80636 München, Germany

**Keywords:** Cardiovascular mechanics, 3D–0D coupling, Inverse problems, Patient-specific modeling, Outcome prediction, Valve regurgitation

## Abstract

Patient-specific computational models of the cardiovascular system can inform clinical decision-making by providing physics-based, non-invasive calculations of quantities that cannot be measured or are impractical to measure and by predicting physiological changes due to interventions. In particular, mixed-dimensional 3D–0D coupled models can represent spatially resolved 3D myocardial tissue mechanics and 0D pressure–flow relationships in heart valves and vascular system compartments, while accounting for their interactions in a closed-loop setting. We present an inverse analysis framework for the automated identification of a set of 3D and 0D patient-specific parameters based on flow, pressure, and cine cardiac MRI measurements. We propose a novel decomposition of the underlying large, nonlinear, and mixed-dimensional inverse problem into an equivalent set of independently solvable, computationally efficient, and well-posed inverse subproblems. This decomposition is enabled by the availability of measurement data of the coupling quantities and ensures a faster convergence toward a unique minimum. The inverse subproblems are solved with a L-BFGS optimization algorithm and an adjoint gradient evaluation. The proposed framework is demonstrated in a clinical case study of an adult repaired tetralogy of Fallot (ToF) patient with severe pulmonary regurgitation. The identified parameters provide a good agreement between measured and computed flows, pressures, and chamber volumes, ensuring a patient-specific model response. The outcome prediction of an in silico pulmonary valve replacement using the personalized model is physiologically consistent and correlates well with postoperative measurements. The proposed framework is essential for developing accurate and reliable cardiovascular digital twins and exploiting their predictive capabilities for intervention planning.

## Introduction

The advancements in medical data acquisition for individual patients and the growing power of computers and algorithms can be combined with our knowledge of physiology and fundamental laws of physics in order to build personalized computational models and assist clinicians in drawing an accurate diagnosis as well as making a reliable prognosis (Corral-Acero et al 2020; Coorey et al 2022; Hose et al 2019; Fumagalli et al 2024; Zingaro et al 2024; Marsden and Esmaily-Moghadam 2015).

Physics-based, law-driven models in the form of governing ordinary and partial differential equations provide a framework to augment clinical and experimental data, enabling the outcome prediction of interventions, even under unseen scenarios (Saltelli 2008; Alber et al 2019; Peirlinck et al 2021; Smith et al 2011; Chapelle and Le Gall 2023; Nordsletten et al 2011; Datz et al 2024; Genet et al 2016). For a reliable postoperative prediction, the model’s accurate representation of the preoperative physiological state is a prerequisite (Spilker and Taylor 2010). A key enabling pillar for the integration of patient data and law-driven computational models is the formulation of well-posed and computationally efficient inverse problems, sometimes referred to as parameter identification or data assimilation methods (Bracamonte et al 2022; Nolte and Bertoglio 2022; Marchesseau et al 2013; Imperiale et al 2021; De Vecchi et al 2016; Bertoglio et al 2014; Molléro et al 2018).

**Fig. 1:**
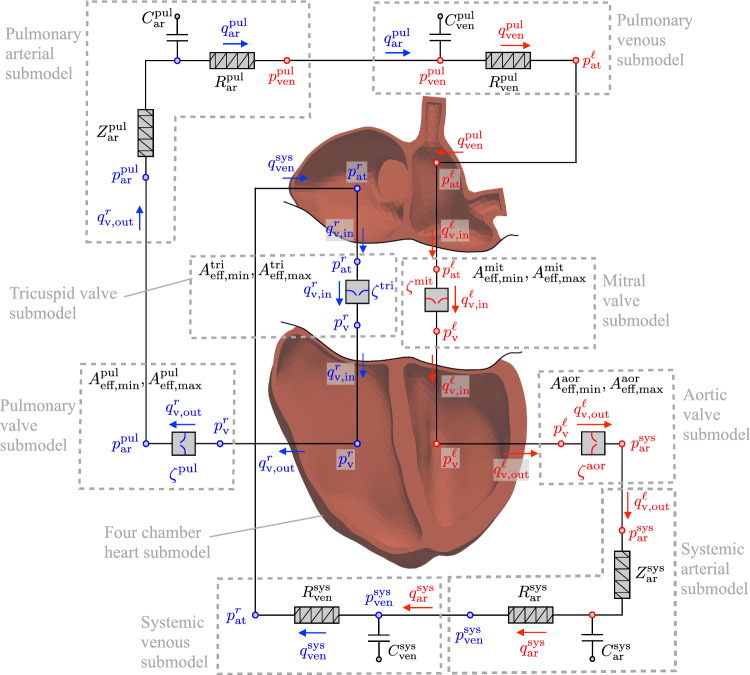
3D–0D coupled closed-loop model of the cardiovascular system. The 3D mesh of the four heart chambers corresponds to the clinical case study of a repaired ToF patient presented in Sect. 3. The separation between atria and ventricles is merely schematic for representing the atrioventricular valves

The decomposition of an inverse problem into several inverse subproblems showed promising results in the context of parameter identification of full 0D closed-loop cardiovascular models (Revie et al 2013; Schiavazzi et al 2017) and 3D–0D abdominal aortic models (Spilker and Taylor 2010). In this work, we propose a novel inverse analysis framework to identify an extensive set of patient-specific parameters of a 3D–0D coupled model of the entire closed-loop cardiovascular system depicted in Fig. 1. In this framework, we decompose the large, nonlinear, and mixed-dimensional inverse problem into several independently solvable and well-posed inverse subproblems.

The proposed decomposition is based on prescribing coupling quantities using the available measurement data while solving the different forward problems independently in a decoupled setting. Despite the decoupled solution strategy, it ensures that the coupling conditions between submodels are always satisfied. Note that each primary variable that appears in more than one submodel in Fig. 1 is a coupling variable. For example, if measurements of the right ventricular and pulmonary arterial pressures, $p_{\textrm{v}}^{r}$ and $p_{\textrm{ar}}^{\textrm{pul}}$, and pulmonary flow $q_{\textrm{v}, \text{ out } }^{r}$ are available, then the parameters of the pulmonary valve submodel can be identified in a completely decoupled manner from neighboring submodels. With the proposed decomposition, we formulate simple and interpretable objective functions and avoid the convergence of the inverse problem to local minima, which do not satisfy all required similarities between computed and measured target quantities.

When building the model, the amount, type, and accuracy of the available measurement data and the relevant physics must be considered (Spilker and Taylor 2010). Complex models with a large number of parameters may deliver a detailed description of some physiological mechanisms. However, they may pose identifiability challenges when trying to compute personalized parameters and reproduce patient-specific responses (Schiavazzi et al 2017; Niederer et al 2019).

In this work, we use anatomic, four-chamber models built with patient-specific geometries based on cine cardiac MRI data. They enable a more accurate representation of atria and their interaction with the ventricles compared to two chamber biventricular models (Peirlinck et al 2021; Hirschvogel et al 2017). We couple the 3D finite element model of the myocardium monolithically with 0D valvular and vascular windkessel models to represent the entire closed-loop cardiovascular system and thereby account for the interaction between all subsystems. (Fedele et al 2023; Strocchi et al 2020; Gerach et al 2021; Pfaller et al 2019). A combination of a passive, anisotropic material model from Holzapfel and Ogden (2009) with active, anisotropic material models (Bestel et al 2001; Sermesant et al 2006) is used for modeling the myocardium. The myofiber orientation for the definition of constitutive anisotropy is generated with rule-based methods (Piersanti et al 2021; Doste et al 2019). The 0D valve model used here is proposed by Mynard et al (2012) and depicts a sophisticated and physically motivated pressure–flow relationship based on the Bernoulli equation, where inertia effects are included, and regurgitation can be represented.

The main advantages of 3D–0D mixed-dimensional models are the consideration of realistic loads that the heart chambers face during contraction and the quantification of clinically relevant integral hemodynamic variables such as blood pressure and flow in the different compartments of the circulatory system (Westerhof et al 2009, 1969; Land et al 2015; Piersanti et al 2022; Regazzoni et al 2022). Additionally, 3D–0D mixed-dimensional models offer a computationally efficient alternative, requiring significantly less time and cost compared to fully spatially resolved 3D fluid dynamics modeling, which is unnecessary for the clinical application at hand. (Caruel et al 2014; Nair et al 2023).

The personalization of computational models based on clinical measurements data represents an inverse problem that can be formulated as an optimization (Nocedal and Wright 1999; Salvador et al 2023; Kehl and Gee 2017; Marsden 2014) or a root-finding problem (Nolte and Bertoglio 2022; Spilker and Taylor 2010; Itu et al 2015; Ismail et al 2013). Bayesian optimization is also widely used in this context(Schiavazzi et al 2017; Lazarus et al 2022; Torbati et al 2024). We utilize here a L-BFGS optimization algorithm combined with an adjoint-based gradient evaluation (Broyden 1970; Fletcher 1970; Goldfarb 1970; Shanno 1970; Alberdi et al 2018; Michaleris et al 1994). We describe the formulation of the underlying adjoint problem and its solution procedure and highlight the particularities of dynamic coupled models. The use of an efficient and exact gradient evaluation method, such as the adjoint method, is crucial for reaching an accurate solution and keeping the computational cost within feasible limits. Therefore, it is widely utilized in the field of cardiovascular mechanics for solving optimization problems (Perego et al 2011; Quarteroni and Rozza 2003; Ismail et al 2013; Oberai et al 2003).

Inverse problem formulations in cardiac mechanics using cine cardiac MRI are described by Xi et al (2013); Genet et al (2014); Rumindo et al (2020), and an overview is given by Bracamonte et al (2022). Imaging techniques such as ultrasound speckle-tracking (Mondillo et al 2011), MRI tissue tagging (Zerhouni et al 1988), and displacement encoding with stimulated echoes (DENSE) (Ghadimi et al 2023; Aletras et al 1999) are designed to measure explicitly displacement field distribution in the tissue and can capture local kinematics. They were, therefore, extensively used in the context of parameter identification (Finsberg et al 2018; Genet et al 2014; Balaban et al 2018; Zhang et al 2020; Asner et al 2016).

Identifying model parameters in hemodynamics is challenging due to three factors: the presence of uncertainty in clinical measurements, the imperfect nature of various assumptions, and the fact that the number of parameters can be quite large compared to the extent of measurements data, especially in models representing the entire circulation (Pant et al 2017). Solving multiple inverse problems for a smaller number of parameters of forward subproblems can reduce the required computational effort and improve the identifiability of the parameters (Pant et al 2017). The importance of the identifiability analysis of an inverse problem is emphasized by Schiavazzi et al (2017) in order to understand whether the parameters can be computed from the available data. Several methods on identifiability are proposed and reviewed in (Miao et al 2011). The compartmental nature of cardiovascular models can be used for the decomposition of a global non-identifiable inverse problem into independently solvable subproblems with identifiable parameters (Schiavazzi et al 2017; Revie et al 2013).

The inverse analysis framework presented here is an extension of our previous work (Hirschvogel et al 2019, 2017). The core novelties are the decomposition of the inverse problem, the use of adjoint-based sensitivities with the L-BFGS optimization, and the calibration to a much richer dataset.

We demonstrate the capabilities of the proposed inverse analysis framework in a retrospective clinical case study of an adult repaired tetralogy of Fallot (ToF) patient who exhibited a pulmonary regurgitation and underwent pulmonary valve replacement. While it has been shown that pulmonary valve replacement improves symptoms and functional status in repaired ToF patients, the optimal timing and indications for the replacement intervention are still debated (Georgiev et al 2020; Rutz et al 2017). Therefore, using a personalized 3D–0D cardiovascular model to support physicians’ decision-making by assessing the preoperative cardiovascular function and predicting its postoperative state might become a valuable clinical instrument.

**Fig. 2:**
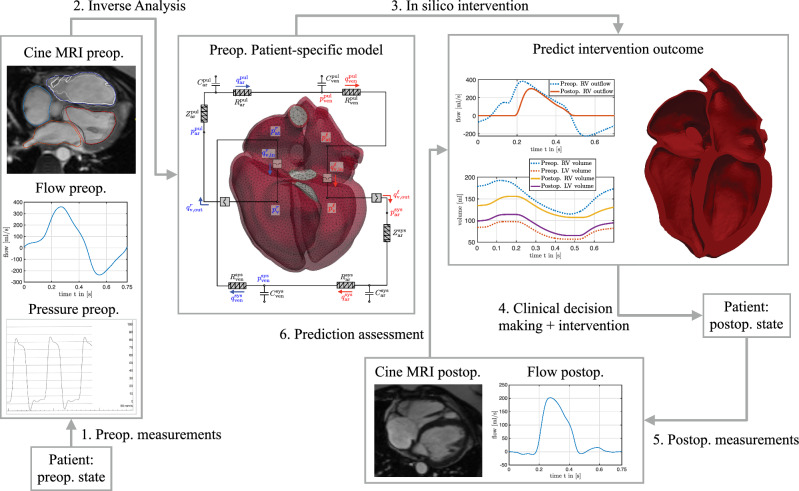
Intervention outcome prediction using a cardiovascular digital twin

The proposed cardiovascular data-informed model can be deployed for an intervention outcome prediction following the steps in Fig. 2. We were able to compute personalized parameters of a high-fidelity model of a ToF patient with manageable computational effort and time for clinical practice. An outcome prediction of a pulmonary valve replacement is performed using the previously calibrated model, but the framework can be easily transferred to other clinical cardiac use cases.

## Methods

### 3D–0D coupled cardiovascular forward model

The 3D–0D coupled closed-loop model of the cardiovascular system considered in this work is an adapted and enhanced version of the model proposed in our previous work by Hirschvogel et al (2017) and is illustrated in Fig. 1. The 0D part is composed of windkessel models of the systemic and pulmonary circulations and Bernoulli valve models of the four heart valves (Westerhof et al 2009; Stergiopulos et al 1999; Mynard et al 2012). The 3D solid mechanics finite element model includes the myocardium of the four heart chambers. All 0D and 3D primary variables, their respective symbols, and units are listed in Table 1.

**Table 1:** Overview of primary variables of the 3D–0D cardiovascular model

0D submodel primary variables		
Variable	Symbol	Unit
Left atrial pressure	$p_{\textrm{at}}^{\ell }$	$[\textrm{kPa}]$
Right atrial pressure	$p_{\textrm{at}}^{r}$	$[\textrm{kPa}]$
Left ventricular pressure	$p_{\textrm{v}}^{\ell }$	$[\textrm{kPa}]$
Right ventricular pressure	$p_{\textrm{v}}^{r}$	$[\textrm{kPa}]$
Systemic arterial pressure	$p_{\textrm{ar}}^{\textrm{sys}}$	$[\textrm{kPa}]$
Systemic venous pressure	$p_{\textrm{ven}}^{\textrm{sys}}$	$[\textrm{kPa}]$
Pulmonary arterial pressure	$p_{\textrm{ar}}^{\textrm{pul}}$	$[\textrm{kPa}]$
Pulmonary venous pressure	$p_{\textrm{ven}}^{\textrm{pul}}$	$[\textrm{kPa}]$
Mitral valve flow	$q_{\textrm{v,in}}^{\ell }$	$[\mathrm {ml \ s^{-1}}]$
Aortic valve flow	$q_{\textrm{v,out}}^{\ell }$	$[\mathrm {ml \ s^{-1}}]$
Tricuspid valve flow	$q_{\textrm{v,in}}^{r}$	$[\mathrm {ml \ s^{-1}}]$
Pulmonary valve flow	$q_{\textrm{v,out}}^{r}$	$[\mathrm {ml \ s^{-1}}]$
Systemic arterial flow	$q_{\textrm{ar}}^{\textrm{sys}}$	$[\mathrm {ml \ s^{-1}}]$
Systemic venous flow	$q_{\textrm{ven}}^{\textrm{sys}}$	$[\mathrm {ml \ s^{-1}}]$
Pulmonary arterial flow	$q_{\textrm{ar}}^{\textrm{pul}}$	$[\mathrm {ml \ s^{-1}}]$
Pulmonary venous flow	$q_{\textrm{ven}}^{\textrm{pul}}$	$[\mathrm {ml \ s^{-1}}]$
Mitral valve state variable	$\zeta ^{\text {mit}}$	$[-]$
Aortic valve state variable	$\zeta ^{\text {aor}}$	$[-]$
Tricuspid valve state variable	$\zeta ^{\text {tri}}$	$[-]$
Pulmonary valve state variable	$\zeta ^{\text {pul}}$	$[-]$

3D submodel primary variables		
Variable	Symbol	Unit
Nodal displacements	$\varvec{d}$	$[\textrm{mm}]$

#### 0D submodels of the vascular system and heart valves

0D windkessel models are widely used to represent the integral pressure–flow relationships in arteries and veins with physiologically interpretable parameters, such as compliance and peripheral resistance (Wetterer 1940; Westerhof et al 2009; Stergiopulos et al 1999). The 3-element and the 2-element windkessel models are utilized here to model the arterial and venous compartments, respectively. In addition to the peripheral resistance $R_{\textrm{ar}}$ and compliance $C_{\textrm{ar}}$, the 3-element windkessel model includes also the characteristic impedance parameter $Z_{\textrm{ar}}$ in order to represent wave travel aspects of the arterial system (Westerhof et al 1969, 2009). The respective governing equations represent reduced dimensional balances of mass and momentum. The equations of the 3-element windkessel models of the systemic and pulmonary arterial compartments are presented for completeness

1 $$\begin{aligned}&C_{\textrm{ar}}^{\textrm{sys}}\left( \frac{\textrm{d} p_{\textrm{ar}}^{\textrm{sys}}}{\textrm{d} t}-Z_{\textrm{ar}}^{\textrm{sys}} \frac{\textrm{d} q_{\textrm{v,out}}^{\ell }}{\textrm{d} t}\right) -q_{\textrm{v,out }}^{\ell }+q_{\textrm{ar}}^{\textrm{sys}}=0, \end{aligned}$$

2 $$\begin{aligned}&\frac{1}{R_{\textrm{ar}}^{\textrm{sys}}}\left( p_{\textrm{ven}}^{\textrm{sys}}-p_{\textrm{ar}}^{\textrm{sys}}+Z_{\textrm{ar}}^{\textrm{sys}} q_{\textrm{v,out}}^{\ell }\right) +q_{\textrm{ar}}^{\textrm{sys}}=0,\end{aligned}$$

3 $$\begin{aligned}&C_{\textrm{ar}}^{\textrm{pul}}\left( \frac{\textrm{d} p_{\textrm{ar}}^{\textrm{pul}}}{\textrm{d} t}-Z_{\textrm{ar}}^{\textrm{pul}} \frac{\textrm{d} q_{\textrm{v,out }}^{r}}{\textrm{d} t}\right) -q_{\textrm{v,out }}^{r}+q_{\textrm{ar}}^{\textrm{pul}}=0, \end{aligned}$$

4 $$\begin{aligned}&\frac{1}{R_{\textrm{ar}}^{\textrm{pul}}}\left( p_{\textrm{ven}}^{\textrm{pul}}-p_{\textrm{ar}}^{\textrm{pul}}+Z_{\textrm{ar}}^{\textrm{pul}} q_{\textrm{v,out}}^{r}\right) +q_{\textrm{ar}}^{\textrm{pul}}=0. \end{aligned}$$

The equations of the 2-element windkessel models of the systemic and pulmonary venous compartments read

5 $$\begin{aligned}&C_{\textrm{ven}}^{\textrm{sys}} \frac{\textrm{d} p_{\textrm{ven}}^{\textrm{sys}}}{\textrm{d} t}-q_{\textrm{ar}}^{\textrm{sys}}+q_{\textrm{ven}}^{\textrm{sys}}=0, \end{aligned}$$

6 $$\begin{aligned}&\frac{1}{R_{\textrm{ven}}^{\textrm{sys}}}\left( p_{\textrm{at}}^r-p_{\textrm{ven}}^{\textrm{sys}}\right) +q_{\textrm{ven}}^{\textrm{sys}}=0, \end{aligned}$$

7 $$\begin{aligned}&C_{\textrm{ven}}^{\textrm{pul}} \frac{\textrm{d} p_{\textrm{ven}}^{\textrm{pul}}}{\textrm{d} t}-q_{\textrm{ar}}^{\textrm{pul}}+q_{\textrm{ven}}^{\textrm{pul}}=0, \end{aligned}$$

8 $$\begin{aligned}&\frac{1}{R_{\textrm{ven}}^{\textrm{pul}}}\left( p_{\textrm{at}}^{\ell }-p_{\textrm{ven}}^{\textrm{pul}}\right) +q_{\textrm{ven}}^{\textrm{pul}}=0. \end{aligned}$$

The 0D valve model proposed by Mynard et al (2012) depicts a physically motivated pressure–flow relationship based on the Bernoulli equation. It includes inertia effects related to blood acceleration. In addition, it involves physiologically interpretable parameters such as the effective orifice area of the valve and can model regurgitation in the form of backward flow. The governing equation reads

9 $$\begin{aligned} \Delta p = \frac{\rho }{2 A_{\textrm{eff}}^{2}} q |q |+ \frac{\rho l_{\textrm{eff}}}{A_{\textrm{eff}}} \frac{\textrm{d} q}{\textrm{d}t}, \end{aligned}$$

where $\Delta p$ is the pressure gradient across the valve, $l_{\textrm{eff}}$ is the effective length, and $\rho$ is the blood density. The effective length $l_{\textrm{eff}}$ represents the length over which the blood accelerates in passing through the valve and was experimentally estimated to be three times the geometric orifice diameter by Flachskampf et al (1993). The effective orifice area (EOA) $A_{\textrm{eff}}$ is expressed as

10 $$\begin{aligned} A_{\textrm{eff}}(t) = \left[ A_{\textrm{eff,max}} - A_{\textrm{eff,min}} \right] \ \zeta (t) + A_{\textrm{eff,min}}, \end{aligned}$$

with $\zeta (t): \ 0 \le \zeta \le 1$. The model parameters $A_{\textrm{eff,min}}$, $A_{\textrm{eff,max}}$ define the minimum and maximum effective orifice areas, respectively. The EOA represents the fluid jet’s minimum cross-sectional area, also called the vena contracta. It should not be confused with the geometric orifice area (GOA), which corresponds to the actual opening area of the valve and can be identified from medical imaging data such as MRI (Garcia and Kadem 2006). The EOA is always equal to or smaller than the GOA and is located some distance after the valve, as fluid streamlines cannot change direction instantaneously (Garcia and Kadem 2006). A valve state variable $\zeta =0$ defines a completely closed state and $\zeta =1$ a fully opened state. It is governed by

11 $$\begin{aligned} \frac{\textrm{d} \zeta }{\textrm{d} t}= {\left\{ \begin{array}{ll}(1-\zeta ) \ K_{\textrm{v o}} \ \Delta p, & \Delta p>0, \\ \zeta \ K_{\textrm{v c}} \ \Delta p, & \Delta p \le 0, \end{array}\right. } \end{aligned}$$

where $K_{\textrm{v o}}$ and $K_{\textrm{v c}}$ define the valve opening and closing rates, respectively. A limitation of this valve model is the assumption that Poiseuille-type viscous losses are small and thus negligible, which is not the case in narrowed valves, where higher flow velocities develop and induce a more significant amount of energy dissipation (Garcia et al 2005).

The model inherently captures isovolumic contraction and relaxation phases through the dynamic valve behavior described in the 0D lumped-parameter circulation model. During these phases, valve states are determined by pressure gradients, ensuring that all valves remain closed, see Equation (11). This prevents volume exchange between chambers and maintains constant ventricular volumes, effectively reproducing the isovolumic conditions observed physiologically.

The overall vector of 0D primary variables, see Fig. 1, is composed of flow, pressure, and valve state variables. It reads

12 $$\begin{aligned} \begin{aligned} \varvec{q} = \Big [&p_{\textrm{at}}^{\ell } \, q_{\textrm{v,in}}^{\ell } \, \zeta ^{\text {mit}} \, q_{\textrm{v,out}}^{\ell } \, \zeta ^{\text {aor}} \, p_{\textrm{v}}^{\ell } \, p_{\textrm{ar}}^{\textrm{sys}} \, q_{\textrm{ar}}^{\textrm{sys}} \, p_{\textrm{ven}}^{\textrm{sys}} \, q_{\textrm{ven}}^{\textrm{sys}} \\&p_{\textrm{at}}^r \, q_{\textrm{v,in}}^r \, \zeta ^{\text {tri}} \, q_{\textrm{v,out}}^r \, \zeta ^{\text {pul}} \, p_{\textrm{v}}^r \, p_{\textrm{ar}}^{\textrm{pul}} \, q_{\textrm{ar}}^{\textrm{pul}} \, p_{\textrm{ven}}^{\textrm{pul}} \, q_{\textrm{ven}}^{\textrm{pul}} \Big ]^{T}. \end{aligned} \end{aligned}$$

The 0D submodel parameters, their respective symbols, and units are listed in Table 2.

**Table 2:** Overview of the 33 parameters of 0D submodels

Parameter	Symbol	Unit
Sys. arterial compliance	$C_{\textrm{ar}}^{\textrm{sys}}$	$[\mathrm {mm^{3} \ kPa^{-1}}]$
Sys. arterial resistance	$R_{\textrm{ar}}^{\textrm{sys}}$	$[\mathrm {kPa \ s \ mm^{-3}}]$
Aor. charac. impedance	$Z_{\textrm{ar}}^{\textrm{sys}}$	$[\mathrm {kPa \ s \ mm^{-3}}]$
Sys. venous compliance	$C_{\textrm{ven}}^{\textrm{sys}}$	$[\mathrm {mm^{3} \ kPa^{-1}}]$
Sys. venous resistance	$R_{\textrm{ven}}^{\textrm{sys}}$	$[\mathrm {kPa \ s \ mm^{-3}}]$
Pul. arterial compliance	$C_{\textrm{ar}}^{\textrm{pul}}$	$[\mathrm {mm^{3} \ kPa^{-1}}]$
Pul. arterial resistance	$R_{\textrm{ar}}^{\textrm{pul}}$	$[\mathrm {kPa \ s \ mm^{-3}}]$
Pul. charac. impedance	$Z_{\textrm{ar}}^{\textrm{pul}}$	$[\mathrm {kPa \ s \ mm^{-3}}]$
Pul. venous compliance	$C_{\textrm{ven}}^{\textrm{pul}}$	$[\mathrm {mm^{3} \ kPa^{-1}}]$
Pul. venous resistance	$R_{\textrm{ven}}^{\textrm{pul}}$	$[\mathrm {kPa \ s \ mm^{-3}}]$
Blood density	$\rho$	$[\mathrm {kg \ m^{-3}}]$
Min. mitral valve EOA	$A_{\textrm{eff,min}}^{\textrm{mit}}$	$[\mathrm {mm^{2}}]$
Max. mitral valve EOA	$A_{\textrm{eff,max}}^{\textrm{mit}}$	$[\mathrm {mm^{2}}]$
Mit. valve opening rate	$K_{\textrm{v o}}^{\textrm{mit}}$	$[\mathrm {s^{-1} \ kPa^{-1}}]$
Mit. valve closing rate	$K_{\textrm{v c}}^{\textrm{mit}}$	$[\mathrm {s^{-1} \ kPa^{-1}}]$
Mit. valve effective length	$l_{\textrm{eff}}^{\textrm{mit}}$	$[\textrm{mm}]$
Min. aortic valve EOA	$A_{\textrm{eff,min}}^{\textrm{aor}}$	$[\mathrm {mm^{2}}]$
Max. aortic valve EOA	$A_{\textrm{eff,max}}^{\textrm{aor}}$	$[\mathrm {mm^{2}}]$
Aor. valve opening rate	$K_{\textrm{v o}}^{\textrm{aor}}$	$[\mathrm {s^{-1} \ kPa^{-1}}]$
Aor. valve closing rate	$K_{\textrm{v c}}^{\textrm{aor}}$	$[\mathrm {s^{-1} \ kPa^{-1}}]$
Aor. valve effective length	$l_{\textrm{eff}}^{\textrm{aor}}$	$[\textrm{mm}]$
Min. tricuspid valve EOA	$A_{\textrm{eff,min}}^{\textrm{tri}}$	$[\mathrm {mm^{2}}]$
Max. tricuspid valve EOA	$A_{\textrm{eff,max}}^{\textrm{tri}}$	$[\mathrm {mm^{2}}]$
Tri. valve opening rate	$K_{\textrm{v o}}^{\textrm{tri}}$	$[\mathrm {s^{-1} \ kPa^{-1}}]$
Tri. valve closing rate	$K_{\textrm{v c}}^{\textrm{tri}}$	$[\mathrm {s^{-1} \ kPa^{-1}}]$
Tri. valve effective length	$l_{\textrm{eff}}^{\textrm{tri}}$	$[\textrm{mm}]$
Min. pulmonary valve EOA	$A_{\textrm{eff,min}}^{\textrm{pul}}$	$[\mathrm {mm^{2}}]$
Max. pulmonary valve EOA	$A_{\textrm{eff,max}}^{\textrm{pul}}$	$[\mathrm {mm^{2}}]$
Pul. valve opening rate	$K_{\textrm{v o}}^{\textrm{pul}}$	$[\mathrm {s^{-1} \ kPa^{-1}}]$
Pul. valve closing rate	$K_{\textrm{v c}}^{\textrm{pul}}$	$[\mathrm {s^{-1} \ kPa^{-1}}]$
Pul. valve effective length	$l_{\textrm{eff}}^{\textrm{pul}}$	$[\textrm{mm}]$

#### 3D solid mechanics submodel of the heart muscle

The patient-specific geometry of the myocardium is obtained by segmenting the respective patient’s cardiac MR images. The selected time snapshot is shortly before atrial systole, also called diastasis. After segmentation, the resulting STL surfaces are used to mesh the initial configuration of the 3D finite element model of the myocardium. No motion is imposed from the imaging data. The subsequent cardiac motion emerges as a result of solving the equations of motion driven by the prescribed active stress.

The continuum mechanical initial boundary value problem of the finite deformation elastodynamics of the myocardium in its weak form is

13 $$\begin{aligned} \begin{aligned}&\int _{\Omega _0} \rho _0 \ddot{\varvec{u}} \cdot \delta \varvec{u} \textrm{d} V+\int _{\Omega _0} \varvec{S}: \delta \varvec{E} \textrm{d} V \\&+\int _{\Gamma _0^{\textrm{R}, \textrm{e}}}\left( \varvec{n}_0 \otimes \varvec{n}_0\right) \left( k_{\textrm{e}} \varvec{u}+c_{\textrm{e}} \dot{\varvec{u}}\right) \cdot \delta \varvec{u} \textrm{d} A \\&+\int _{\Gamma _0^{\textrm{R,ves}}}\left( k_{\textrm{ves}} \varvec{u}+c_{\textrm{ves}} \dot{\varvec{u}}\right) \cdot \delta \varvec{u} \textrm{d} A \\&+ \sum _{c=\textrm{at}, \textrm{v}} \sum _{i=\ell , r} \int _{\Gamma _{0, c}^{\textrm{0D}, i}} p_{c}^i J \varvec{F}^{-\textrm{T}} \varvec{n}_0 \cdot \delta \varvec{u} \textrm{d} A=0, \; \forall \delta \varvec{u}, \end{aligned} \end{aligned}$$

where $\varvec{u}$ is the displacement field, $\varvec{F}$ is the deformation gradient, $J = \text {det}(\varvec{F})$ is its determinant, $\varvec{E} = \frac{1}{2}(\varvec{F}^{T}\varvec{F} - \varvec{I})$ is the Green–Lagrange strain tensor, $\varvec{S}$ is the second Piola–Kirchhoff stress tensor, and $\delta \varvec{u}$ is the test function. The 3D domain of the segmented initial configuration is $\Omega _0$, and its reference density is $\rho _0$. Note that the segmented, initial configuration at $t_0$, the start of atrial contraction, is not stress-free (Hadjicharalambous et al 2021). Therefore, a prestressing step is performed using the method proposed by Gee et al (2010). The mechanical interaction of the heart with the surrounding tissue at the epicardium is modeled with a viscoelastic boundary condition on $\Gamma _0^{\textrm{R}, \textrm{e}}$ using the stiffness $k_\textrm{e}$ and the viscosity $c_\textrm{e}$, which are assumed to act only in initial surface normal direction $\varvec{n}_0$. The pressures $p_{c}^{i}$ within the heart chambers act as boundary traction forces in current normal direction onto the respective endocardial inner surfaces $\Gamma _{0, \textrm{v}}^{\textrm{0D}, i}$ and $\Gamma _{0, \textrm{at}}^{\textrm{0D}, i}$, where $i = r, \ell$. It contributes, therefore, to the external virtual work, as can be seen in Equation (13). The stress tensor $\varvec{S}$ is composed additively of a hyperelastic passive part and an active contribution as

14 $$\begin{aligned} \varvec{S} = \dfrac{\partial \Psi }{\partial \varvec{E}} + \tau _a(t) \varvec{f}_0 \otimes \varvec{f}_0, \end{aligned}$$

where $\Psi$ denotes the strain energy density function proposed by Holzapfel and Ogden (2009) for the passive material behavior of myocardial tissue and reads

15 $$\begin{aligned} \begin{aligned} \Psi =&\frac{a}{2 b}\left( e^{b\left( \bar{I}_{C}-3\right) }-1\right) +\sum _{i=f, s} \frac{a_i}{2 b_i}\left( e^{b_i\left( IV_{i}-1\right) ^2}-1\right) \\&+\frac{a_{f s}}{2 b_{f s}}\left( e^{b_{f s} VIII_{f s}^2}-1\right) +\frac{\kappa }{2}(J-1)^2. \end{aligned} \end{aligned}$$

This material model is orthotropic, nonlinearly elastic, and incompressible (Holzapfel and Ogden 2009). It distinguishes locally within the 3D myocardium between three mutually orthogonal directions. The local coordinate system consists of the muscle fiber axis $\varvec{f}_0$, the sheet axis $\varvec{s}_0$, and the wall-normal axis $\varvec{n}_0$. The model parameters were fitted to experimental data stemming from shear and biaxial tests and are kept fixed here (Holzapfel and Ogden 2009).

**Fig. 3:**
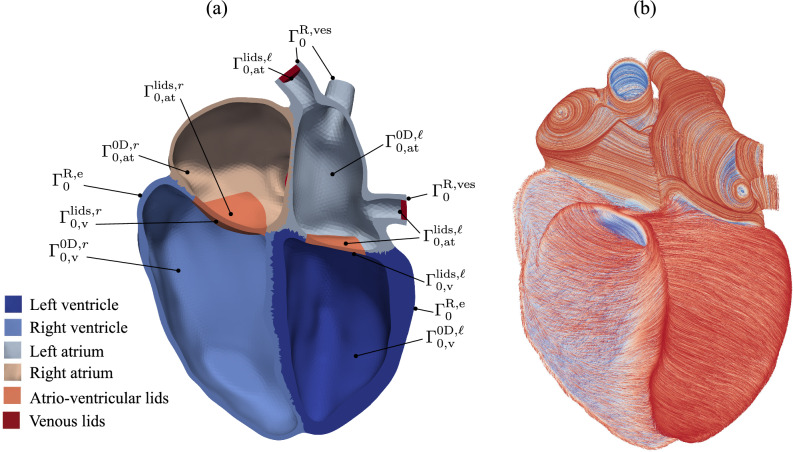
**a** Mesh partition and surface boundaries definition used in Eqs. (13) and (26). **b** Rule-based fiber orientation (Piersanti et al 2021)

The time dependent active stress $\tau _a(t)$ acts along the myocardial muscle fiber direction and stems from the ODE proposed by Sermesant et al (2006); Bestel et al (2001)

16 $$\begin{aligned} \dot{\tau }_{\textrm{a}}=-|u |\tau _{\textrm{a}}+ \sigma _0 |u |_{+}, \end{aligned}$$

with |*u*| for the absolute value of *u* and $|u |_{+}:= \text {max}(0,u)$. The transmembrane potential *u* is built by scaling a normalized activation function $\hat{f}(t) \in [0,1]$ with the active stress upstroke and relaxation rates $\alpha _{max}$ and $\alpha _{min}$, respectively

17 $$\begin{aligned} u=\hat{f}(t) \cdot \alpha _{\max }+(1-\hat{f}(t)) \cdot \alpha _{\min }. \end{aligned}$$

The normalized activation function is

18 $$\begin{aligned} \begin{aligned} \hat{f}(t)=&\left( K\left( t-c_1\right) +1\right) \cdot \mathcal {H}\left[ K\left( t-c_1\right) +1\right] \\&-K\left( t-c_1\right) \cdot \mathcal {H}\left[ K\left( t-c_1\right) \right] \\&-K\left( t-c_2\right) \cdot \mathcal {H}\left[ K\left( t-c_2\right) \right] \\&+\left( K\left( t-c_2\right) -1\right) \cdot \mathcal {H}\left[ K\left( t-c_2\right) -1\right] , \end{aligned} \end{aligned}$$

with

19 $$\begin{aligned} c_1=t_{\text{ contr } }+\frac{\alpha _{\max }}{K\left( \alpha _{\max }-\alpha _{\min }\right) }, \end{aligned}$$

and

20 $$\begin{aligned} c_2=t_{\text{ relax } }-\frac{\alpha _{\max }}{K\left( \alpha _{\max }-\alpha _{\min }\right) }, \end{aligned}$$

and $\mathcal {H}\left[ \cdot \right]$ the Heaviside function. Throughout this work, the regularization parameter $K=10$ is chosen. $t_{\text{ contr } }$ and $t_{\text{ relax } }$ define the start time of contraction and relaxation, respectively. In this model, all ventricular and atrial elements are activated simultaneously, respectively. The brief delay associated with the propagation of action potentials through the myocardial syncytium is not represented. Instead, we assume that spatially homogeneous activation provides a sufficient approximation for the cardiac dynamics under investigation. The 3D submodel parameters are listed in Table 3.

**Table 3:** Overview of the 38 parameters of the 3D submodel

Parameter	Symbol	Unit
Left ventr. upstroke rate	$\alpha _{\textrm{max,v}}^{\ell }$	$[\mathrm {s^{-1}}]$
Left ventr. relaxation rate	$\alpha _{\textrm{min,v}}^{\ell }$	$[\mathrm {s^{-1}}]$
Left ventr. contractility	$\sigma _{\textrm{0,v}}^{\ell }$	$[\textrm{kPa}]$
Right ventr. upstroke rate	$\alpha _{\textrm{max,v}}^{r}$	$[\mathrm {s^{-1}}]$
Right ventr. relaxation rate	$\alpha _{\textrm{min,v}}^{r}$	$[\mathrm {s^{-1}}]$
Right ventr. contractility	$\sigma _{\textrm{0,v}}^{r}$	$[\textrm{kPa}]$
Ventr. contraction start time	$t_{\textrm{contr,v}}$	$[\textrm{s}]$
Ventr. relaxation start time	$t_{\textrm{relax,v}}$	$[\textrm{s}]$
Left atrial upstroke rate	$\alpha _{\textrm{max,at}}^{\ell }$	$[\mathrm {s^{-1}}]$
Left atrial relaxation rate	$\alpha _{\textrm{min,at}}^{\ell }$	$[\mathrm {s^{-1}}]$
Left atrial contractility	$\sigma _{\textrm{0,at}}^{\ell }$	$[\textrm{kPa}]$
Right atrial upstroke rate	$\alpha _{\textrm{max,at}}^{r}$	$[\mathrm {s^{-1}}]$
Right atrial relaxation rate	$\alpha _{\textrm{min,at}}^{r}$	$[\mathrm {s^{-1}}]$
Right atrial contractility	$\sigma _{\textrm{0,at}}^{r}$	$[\textrm{kPa}]$
Atrial contraction start time	$t_{\textrm{contr,at}}$	$[\textrm{s}]$
Atrial relaxation start time	$t_{\textrm{relax,at}}$	$[\textrm{s}]$
Duration of a cardiac cycle	$T_{\textrm{cycl}}$	$[\textrm{s}]$
Stiffness at surface boundaries	$k_{\textrm{e}}$, $k_{\textrm{ves}}$	$[\mathrm {kPa \ mm^{-1}}]$
Viscosity at surface boundaries	$c_{\textrm{e}}$, $c_{\textrm{ves}}$	$[\mathrm {kPa \ s \ mm^{-1}}]$
Passive myocardial material (Holzapfel and Ogden 2009)	*a*, $a_f$	$[\textrm{kPa}]$
	$a_s$, $a_{fs}$	$[\textrm{kPa}]$
	*b*, $b_f$	$[-]$
	$b_s$, $b_{fs}$	$[-]$
Incomp. penalty parameter	$\kappa$	$[\textrm{kPa}]$
Mass proportional damping	$c_{M}$	$[\mathrm {s^{-1}}]$
Stiffness proportional damping	$c_{K}$	$[\textrm{s}]$
Myocardial reference density	$\rho _0$	$[\mathrm {kg \ mm^{-3}}]$

#### Coupling conditions

The 0D and 3D submodels are coupled via dynamic and kinematic coupling conditions. The dynamic coupling is associated with the external forces applied on the inner endorcardial surfaces $\Gamma _{0, c}^{\textrm{0D}, i}$ of 3D heart chambers in the normal direction. These external forces are proportional to 0D chamber pressure variables $p_{c}^i$. Their contribution to the external virtual work reads

21 $$\begin{aligned} {\delta \mathcal {W}_{\textrm{ext}}} = - \sum _{c=\textrm{at}, \textrm{v}} \sum _{i=\ell , r} \int _{\Gamma _{0, c}^{\textrm{0D}, i}} p_{c}^i J \varvec{F}^{-\textrm{T}} \varvec{n}_0 \cdot \delta \varvec{u} \textrm{d} A, \end{aligned}$$

and is depicted in the last term of the weak form (13). The conservation of mass in the heart chambers constitutes the kinematic coupling condition between 0D and 3D submodels and reads

22 $$\begin{aligned}&\frac{\textrm{d} V_{\textrm{at}}^{\ell }(\varvec{u})}{\textrm{d} t} - q_{\textrm{ven}}^{\textrm{pul}} + q_{\textrm{v,in}}^{\ell } = 0, \end{aligned}$$

23 $$\begin{aligned}&\frac{\textrm{d} V_{\textrm{v}}^{\ell }(\varvec{u})}{\textrm{d} t} - q_{\textrm{v,in}}^{\ell } + q_{\textrm{v,out}}^{\ell } = 0, \end{aligned}$$

24 $$\begin{aligned}&\frac{\textrm{d} V_{\textrm{at}}^{r}(\varvec{u})}{\textrm{d} t} - q_{\textrm{ven}}^{\textrm{sys}} + q_{\textrm{v,in}}^{r} = 0, \end{aligned}$$

25 $$\begin{aligned}&\frac{\textrm{d} V_{\textrm{v}}^{r}(\varvec{u})}{\textrm{d} t} - q_{\textrm{v,in}}^{r} + q_{\textrm{v,out}}^{r} = 0, \end{aligned}$$

where the current volume $V_{c}^{i}(\varvec{u})$ enclosed in a heart chamber is computed as boundary integral over its endocardial surface using the displacement field $\varvec{u}$

26 $$\begin{aligned} \begin{aligned} V_c^i(\varvec{u})&= \frac{1}{3} \int _{\Gamma _c^{0 \textrm{D}, i} \cup \Gamma _c^{\textrm{lids}, i}} \varvec{x} \cdot \varvec{n} \mathrm {~d} a \\&= \frac{1}{3} \int _{\Gamma _{0, c}^{0 \textrm{D}, i} \cup \Gamma _{0, c}^{\textrm{lids}, i}}\left( \varvec{u}+\varvec{x}_0\right) \cdot J \varvec{F}^{-\textrm{T}} \varvec{n}_0 \mathrm {~d} A, \end{aligned} \end{aligned}$$

with $i=\ell , r$ and $c=\textrm{v}, \textrm{at}$. The vectors $\varvec{x}$ and $\varvec{x}_0$ define the current and initial configuration, respectively. Note that artificial lids are added to the anatomic 3D heart model in the positions of the four valves as well as at the interface between atria and veins, as shown in Fig. 3, in order to enclose the volumes of the heart chambers and be able to evaluate Equation (26).

#### Discretization and solution

The governing equations of the 0D submodels of the vascular system and heart valves are discretized in time with the One-Step-$\theta$ scheme (Barclay et al 2000). The resulting time-discrete residual vector $\varvec{R}^{\mathrm{{0D}}}_{k+1}$ is depicted in appendix A in Equation (A).

The finite element discretization of the 3D domain $\Omega _0$ is performed with tetrahedral elements. The space-discrete equation of motion of the 3D submodel reads

27 $$\begin{aligned} \begin{aligned}&\varvec{M} \ddot{\varvec{d}}+\varvec{C} \dot{\varvec{d}}+\varvec{F}_{\textrm{int}}(\varvec{d}\, )-\varvec{F}_{\textrm{ext}}(\varvec{d},\dot{\varvec{d}},\varvec{q})=\varvec{0}, \\&\text {in} \quad \bar{\Omega }_0 \times [0, T_{cycl}], \end{aligned} \end{aligned}$$

where $\varvec{d}$ denotes the vector of discrete nodal displacements and $\bar{\Omega }_0$ the space-discretized domain. The mass matrix $\varvec{M}$ and damping matrix $\varvec{C}$ are chosen to be constant, while the internal force vector $\varvec{F}_{\textrm{int}}$ is a nonlinear function of the nodal displacements $\varvec{d}$. The external force vector $\varvec{F}_{\textrm{ext}}$ depends not only on the nodal displacements $\varvec{d}$ and velocities $\dot{\varvec{d}}$ but also on the pressures of the four heart chambers, which are part of the 0D primary variables $\varvec{q}$. The Rayleigh damping matrix is defined as

28 $$\begin{aligned} \varvec{C}= c_{M} \ \varvec{M} + c_{K} \ \frac{\partial \varvec{F}_{\textrm{int}}}{\partial \varvec{d}}\bigg |_{\varvec{d}=\varvec{0}}. \end{aligned}$$

The velocity vector $\varvec{v} = \dot{\varvec{d}}$ and acceleration vector $\varvec{a} = \ddot{\varvec{d}}$ in the nonlinear system of Equations (27) are discretized in time with the generalized-$\alpha$ scheme (Chung and Hulbert 1993). The residual vector is evaluated at as

29 $$\begin{aligned} \begin{aligned} \varvec{R}^{\mathrm{{3D}}}_{k+1} =&\varvec{M}\ \varvec{a}_{k+1-\alpha _m} + \varvec{C}\ \varvec{v}_{k+1-\alpha _f} \\&+ \varvec{F}_{\textrm{int},k+1-\alpha _f} - \varvec{F}_{\textrm{ext},k+1-\alpha _f} = \varvec{0}, \end{aligned} \end{aligned}$$

where $(\bullet )_{k+1-\alpha _{[\circ ]}}=\left( 1-\alpha _{[\circ ]}\right) (\bullet )_{k+1}+\alpha _{[\circ ]}(\bullet )_k$ and $\alpha _m,\alpha _f \in [0,1]$. The nonlinear system of coupled Equations (A) and (29) is solved iteratively with a monolithic Newton–Raphson scheme and a consistent linearization (Hirschvogel et al 2017). The computation is summarized in Algorithm 1. Since the dynamics of the coupled model are driven by periodic active stress functions, the solution also has to be periodic independently from the initial conditions. Therefore, the cardiac cycle, with a starting time of $t_0$ and a duration of $T_{\textrm{cycl}}$, is recomputed with updated initial conditions stemming from the last time step of the previous cycle until the periodicity criterion

30 $$\begin{aligned} \begin{aligned} E_{\textrm{cycl}}=&\max \left\{ \left| \frac{p_{\textrm{ar}}^{\textrm{sys}}\left( t_0+T_{\textrm{cycl}}\right) -p_{\textrm{ar}}^{\textrm{sys}}\left( t_0\right) }{p_{\textrm{ar}}^{\textrm{sys}}\left( t_0\right) }\right| ,\right. \\&\left| \frac{p_{\textrm{ar}}^{\textrm{pul}}\left( t_0+T_{\textrm{cycl}}\right) -p_{\textrm{ar}}^{\textrm{pul}}\left( t_0\right) }{p_{\textrm{ar}}^{\textrm{pul}}\left( t_0\right) }\right| , \\&\left| \frac{p_{\textrm{ven}}^{\textrm{sys}}\left( t_0+T_{\textrm{cycl}}\right) -p_{\textrm{ven}}^{\textrm{sys}}\left( t_0\right) }{p_{\textrm{ven}}^{\textrm{sys}}\left( t_0\right) }\right| , \\&\left| \frac{p_{\textrm{ven}}^{\textrm{pul}}\left( t_0+T_{\textrm{cycl}}\right) -p_{\textrm{ven}}^{\textrm{pul}}\left( t_0\right) }{p_{\textrm{ven}}^{\textrm{pul}}\left( t_0\right) }\right| , \\&\left| \frac{V_{\textrm{v}}^{\ell }\left( t_0+T_{\textrm{cycl}}\right) -V_{\textrm{v}}^{\ell }\left( t_0\right) }{V_{\textrm{v}}^{\ell }\left( t_0\right) }\right| , \\&\left. \left| \frac{V_{\textrm{v}}^r\left( t_0+T_{\textrm{cycl}}\right) -V_{\textrm{v}}^r\left( t_0\right) }{V_{\textrm{v}}^r\left( t_0\right) }\right| , \right. \\&\left| \frac{V_{\textrm{at}}^r\left( t_0+T_{\textrm{cycl}}\right) -V_{\textrm{at}}^r\left( t_0\right) }{V_{\textrm{at}}^r\left( t_0\right) }\right| , \\&\left. \left| \frac{V_{\textrm{at}}^r\left( t_0+T_{\textrm{cycl}}\right) -V_{\textrm{at}}^r\left( t_0\right) }{V_{\textrm{at}}^r\left( t_0\right) }\right| \right\} \le \epsilon _{\textrm{cycl}}, \end{aligned} \end{aligned}$$

is met, where $\epsilon _{\textrm{cycl}}$ is the periodicity error tolerance.

**Algorithm 1:**
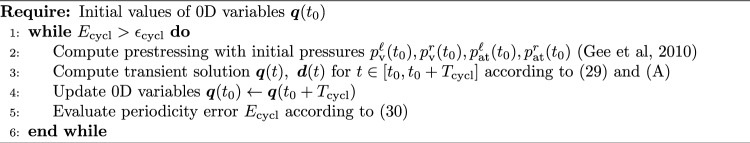
3D–0D coupled forward problem of the cardiovascular dynamics

### Clinical measurements

**Fig. 4:**
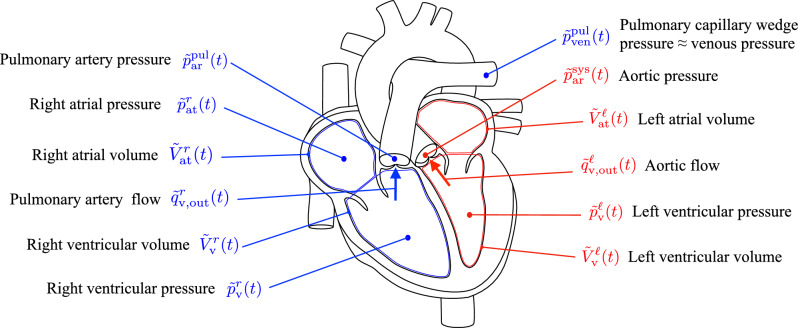
Overview of clinical measurements

The clinical measurements considered here and visualized in Fig. 4 are usually performed by clinicians on repaired ToF patients. They are acquired with cine cardiac MRI, phase-contrast MRI flow measurement, and catheterization-based pressure measurement.

The cine MRI data are acquired in the axial direction with 6 mm slice thickness, at 25 equidistant time points within the cardiac cycle, and an acquisition matrix of $256 \times 256$ (Fratz et al 2009; D’Alto et al 2016). The segmentation of endocardial and epicardial contours provides the 3D anatomic geometry of the myocardium and the temporal evolution of heart chamber volumes during the cardiac cycle. Geometric orifice areas of atrioventricular valves are identified from cine cardiac MRI data by cardiac radiology experts.

The flow through the aorta and pulmonary artery is measured with 2D phase-contrast MRI with a temporal resolution of 30 per cardiac cycle Meierhofer et al (2015); Wymer et al (2020). The discrete volume and flow measurements are interpolated with the cubic spline data interpolation function in MATLAB (MATLAB 2022).

The left and right ventricular, aortic, pulmonary arterial, right atrial, and pulmonary venous pressures are measured as a function of time during several cardiac cycles using a balloon-tipped, multi-lumen catheter (Del Rio-Pertuz et al 2023).

In order to differentiate between measured and computed quantities throughout this work, measured quantities are marked with a tilde symbol $\left( \ \tilde{\cdot } \ \right)$.

### Inverse analysis framework

Patient-specific parameters of 0D and 3D submodels are identified by solving a set of inverse subproblems listed in Table 4 based on the clinical measurements presented in Sect. 2.2. They thereby minimize the difference between measured and computed values of target quantities.

Our novel approach is based on the availability of clinical measurements of coupling quantities between submodels. The availability of these data enables the decomposition of the global overall inverse problem of identifying 0D model parameters listed in Table 4 into several well-posed, computationally efficient, and independently solvable inverse subproblems. Within each 0D inverse subproblem, only the corresponding submodel is used as the forward problem in a decoupled, open-loop setting. For example, since measurements of the right ventricular and pulmonary arterial pressures, $\tilde{p}_{\textrm{v}}^{r}$ and $\tilde{p}_{\textrm{ar}}^{\textrm{pul}}$, and pulmonary flow $\tilde{q}_{\textrm{v,out }}^{r}$ are available, the parameters of the pulmonary valve submodel, $A_{\textrm{eff,max}}^{\textrm{pul}}$ and $A_{\textrm{eff,min}}^{\textrm{pul}}$, can be identified in a completely decoupled manner from other neighboring submodels. All primary variables and coupling relationships can be visualized in Fig. 1. Prescribing the measurement of the coupling quantity $\tilde{q}_{\textrm{v,out }}^{r}$, for instance, in both the pulmonary valve and pulmonary arterial windkessel inverse subproblems satisfies implicitly the corresponding coupling condition.

The active stress parameters of the 3D four-chamber submodel are then identified in a subsequent inverse subproblem using the entire 3D–0D coupled closed-loop model as the forward problem with the already identified 0D parameter values being fixed; see last row of Table 4. Considering a decoupled 3D submodel as the forward problem in this latter step is unnecessary, given that solving 0D submodels is computationally negligible compared to solving the 3D submodel. Additionally, we do not dispose of the measurements of all coupling quantities involved in the 3D submodel, such as the left atrial pressure and flows through atrioventricular valves.

The proposed decoupled framework ensures a unique solution and a faster convergence compared to the solution of one single global inverse problem for all 3D and 0D parameters. In addition, it avoids the propagation of parameter identification errors from one submodel to the others by directly prescribing coupling quantities based on their measurements when solving forward problems.

Only the independent parameters to be identified via inverse analysis are listed in Table 4. The other parameters listed in the extensive Tables 2 and 3 are either dependent on the identified ones or generic, and their definition method will be discussed in the following subsections.

**Table 4:** Overview over the inverse subproblems for the identification of independent patient-specific parameters of the 3D–0D cardiovascular model. The 0D inverse subproblems are solved independently from each other in a first step, while the 3D inverse subproblem is solved in a subsequent step with the identified 0D parameters being fixed

Inverse subproblem	Parameters to be identified	Forward problem	Output	Required measurements	Objective function
*Identification of 0D submodel parameters*					
Eq. (31)	$C_{\textrm{ar}}^{\textrm{sys}}$, $R_{\textrm{ar}}^{\textrm{sys}}$, $Z_{\textrm{ar}}^{\textrm{sys}}$	Eq. (33)	$p_{\textrm{ar}}^{\textrm{sys}}(t)$	$\tilde{p}_{\textrm{ar}}^{\textrm{sys}}(t)$, $\tilde{p}_{\textrm{ven}}^{\textrm{sys}}(t)$, $\tilde{q}_{\textrm{v,out}}^{\mathrm {\ell }}(t)$	Eq. (32)
Eq. (31)	$C_{\textrm{ar}}^{\textrm{pul}}$, $R_{\textrm{ar}}^{\textrm{pul}}$, $Z_{\textrm{ar}}^{\textrm{pul}}$	Eq. (33)	$p_{\textrm{ar}}^{\textrm{pul}}(t)$	$\tilde{p}_{\textrm{ar}}^{\textrm{pul}}(t)$, $\tilde{p}_{\textrm{ven}}^{\textrm{pul}}(t)$, $\tilde{q}_{\textrm{v,out}}^{\textrm{r}}(t)$	Eq. (32)
Eq. (38)	$A_{\textrm{eff,max}}^{\textrm{aor}}$, $A_{\textrm{eff,min}}^{\textrm{aor}}$	Eq. (44, 46)	$q_{\textrm{v,out}}^{\mathrm {\ell }}(t)$	$\tilde{q}_{\textrm{v,out}}^{\mathrm {\ell }}(t)$, $\tilde{p}_{\textrm{v}}^{\ell }(t)$, $\tilde{p}_{\textrm{ar}}^{\mathrm{{sys}}}(t)$	Eq. (39)
Eq. (38)	$A_{\textrm{eff,max}}^{\textrm{pul}}$, $A_{\textrm{eff,min}}^{\textrm{pul}}$	Eq. (44, 46)	$q_{\textrm{v,out}}^{r}(t)$	$\tilde{q}_{\textrm{v,out}}^{r}(t)$, $\tilde{p}_{\textrm{v}}^{r}(t)$, $\tilde{p}_{\textrm{ar}}^{\mathrm{{pul}}}(t)$	Eq. (39)
*Identification of 3D submodel parameters*					
Eq. (49)	$\varvec{\phi }$, Eq. (50)	Eq. (29, A)	$\varvec{d}(t)$	$\tilde{V}_{\textrm{v}}^{\mathrm {\ell }}(t)$, $\tilde{V}_{\textrm{v}}^{r}(t)$, $\tilde{V}_{\textrm{at}}^{\mathrm {\ell }}(t)$, $\tilde{V}_{\textrm{at}}^{r}(t)$	Eq. (51)

#### Identification of 0D submodels parameters

For the identification of arterial compliance, resistance, and characteristic impedance parameters, we formulate an unconstrained minimization problem of a scalar-valued objective function

31 $$\begin{aligned} \mathop {\textrm{argmin}}\limits _{\varvec{\phi }_{\textrm{ar}}} f_{\textrm{ar}}(\varvec{\phi }_{\textrm{ar}}), \; \text {with} \; \varvec{\phi }_{\textrm{ar}} =\left[ R_{\textrm{ar}}, C_{\textrm{ar}}, Z_{\textrm{ar}} \right] , \end{aligned}$$

where the superscripts $(\cdot )^{\textrm{sys}}$ and $(\cdot )^{\textrm{pul}}$ are omitted, since the same approach is adopted for both systemic and pulmonary arterial windkessel parameters. The objective function is

32 $$\begin{aligned} \begin{aligned} f_{\textrm{ar}}(\varvec{\phi }_{\textrm{ar}})&= \frac{\left[ \text {max}(p_{\textrm{ar}}(\varvec{\phi }_{\textrm{ar}},t))-\text {max}(\tilde{p}_{\textrm{ar}}(t)) \right] ^{2}}{\text {max}(\tilde{p}_{\textrm{ar}}(t))^{2}} \\&+ \frac{\left[ \text {min}(p_{\textrm{ar}}(\varvec{\phi }_{\textrm{ar}},t))-\text {min}(\tilde{p}_{\textrm{ar}}(t)) \right] ^{2}}{\text {max}(\tilde{p}_{\textrm{ar}}(t))^{2}} \\&+ \frac{\left[ p_{\textrm{ar}}(\varvec{\phi }_{\textrm{ar}},t_{0}+T_{cycl}) - \tilde{p}_{\textrm{ar}}(t_{0}+T_{cycl}) \right] ^{2}}{\text {max}(\tilde{p}_{\textrm{ar}}(t))^{2}} \\&+ \frac{\left[ \text {max}(\dot{p}_{\textrm{ar}}(\varvec{\phi }_{\textrm{ar}},t))-\text {max}(\tilde{\dot{p}}_{\textrm{ar}}(t)) \right] ^{2}}{\text {max}(\tilde{\dot{p}}_{\textrm{ar}}(t))^{2}}, \end{aligned} \end{aligned}$$

subject to the 3-element windkessel equation

33 $$\begin{aligned} \begin{aligned}&C_{\textrm{ar}} R_{\textrm{ar}} \frac{\textrm{d} p_{\textrm{ar}}}{\textrm{d} t} + p_{\textrm{ar}} - \tilde{p}_{\textrm{ven}} - \left( R_{\textrm{ar}} + Z_{\textrm{ar}} \right) \tilde{q}_{\textrm{v,out}} \\&- Z_{\textrm{ar}} C_{\textrm{ar}} R_{\textrm{ar}} \frac{\textrm{d} \tilde{q}_{\textrm{v,out}}}{\textrm{d} t} = 0, \end{aligned} \end{aligned}$$

with $t \in [t_0, t_0+T_{cycl}]$ and the initial condition $p_{\textrm{ar}}(t_0) = \tilde{p}_{\textrm{ar}}(t_0)$, taken from the respective clinical measurement $\tilde{p}_{\textrm{ar}}(t)$. The operators $\text {max}( p (t)) = \text {max} \{ p(t): t \in [t_0, t_0+T_{cycl}] \}$ and $\text {min}( p (t)) = \text {min} \{ p (t): t \in [t_0, t_0+T_{cycl}] \}$ in Equation (32) define the maximum and minimum values of a pressure function *p*(*t*), respectively. The minimization of the first and second terms of the objective function $f_{\textrm{ar}}(\varvec{\phi }_{\textrm{ar}})$ reduces the difference between measured and computed values of the maximum and minimum arterial pressures, respectively. Pressure extrema values have been used in this context extensively in literature (Ismail et al 2013; Spilker and Taylor 2010; Revie et al 2013). The third term reduces the difference between measured and computed pressure values at the end of the cardiac cycle $t=t_0+T_{cycl}$ to enforce periodicity. The fourth term in Equation (32) enforces similarity between the computed maximum systolic pressure rate and its measured counterpart.

Note that Equation (33) is derived by combining (1) and (2) and eliminating the outlet flow variable $q_{\textrm{ar}}$, since only the pressure $p_{\textrm{ar}}$ is of interest for the evaluation of objective function (32). The venous pressure $\tilde{p}_{\textrm{ven}}(t)$ and the inlet flow through the respective semilunar valve $\tilde{q}_{\textrm{v,out}}(t)$ are prescribed quantities based on clinical data. Thus, (33) is decoupled from other submodels and can be solved independently for $p_{\textrm{ar}}$.

The systemic and pulmonary venous resistances $R^{\mathrm{{sys}}}_{\textrm{ven}}$ and $R^{\mathrm{{pul}}}_{\textrm{ven}}$ as well as compliances $C^{\mathrm{{sys}}}_{\textrm{ven}}$ and $C^{\mathrm{{pul}}}_{\textrm{ven}}$ are not identified as independent optimization variables using an inverse problem as their arterial counterparts, as the required pressure and flow measurements cannot be performed. Therefore, we propose to identify them based on the following relationships with their arterial counterparts

34 $$\begin{aligned}&R^{\mathrm{{sys}}}_{\textrm{ven}} = \frac{1}{10} \ R^{\mathrm{{sys}}}_{\textrm{ar}}, \end{aligned}$$

35 $$\begin{aligned}&C^{\mathrm{{sys}}}_{\textrm{ven}} = 30 \cdot C^{\mathrm{{sys}}}_{\textrm{ar}}, \end{aligned}$$

36 $$\begin{aligned}&R^{\mathrm{{pul}}}_{\textrm{ven}} = R^{\mathrm{{pul}}}_{\textrm{ar}},\end{aligned}$$

37 $$\begin{aligned}&C^{\mathrm{{pul}}}_{\textrm{ven}} = 2.5 \cdot C^{\mathrm{{pul}}}_{\textrm{ar}}, \end{aligned}$$

which were derived in literature data and experimental studies (Gelman et al 2008; Wang et al 2006; Young 2010; Gaar Ka et al 1967; Tanaka et al 1986). For the identification of the maximum and minimum effective orifice area parameters $A_{\textrm{eff,max}}$ and $A_{\textrm{eff,min}}$ of a Bernoulli valve model (9-11), we formulate the following minimization problem of the scalar-valued objective function $f_{\textrm{val}}(\varvec{\phi }_{\textrm{val}})$

38 $$\begin{aligned} \mathop {\textrm{argmin}}\limits _{\varvec{\phi }_{\textrm{val}}} f_{\textrm{val}}(\varvec{\phi }_{\textrm{val}}), \; \text {with} \; \varvec{\phi }_{\textrm{val}} =\left[ A_{\textrm{eff,max}}, A_{\textrm{eff,min}} \right] , \end{aligned}$$

and

39 $$\begin{aligned} \begin{aligned} f_{\textrm{val}}(\varvec{\phi }_{\textrm{val}}) =&\left[ V_{\text {for}} (\varvec{\phi }_{\textrm{val}}) - \tilde{V}_{\text {for}} \right] ^{2} \\&+ \left[ V_{\text {back}} (\varvec{\phi }_{\textrm{val}}) - \tilde{V}_{\text {back}} \right] ^{2}, \end{aligned} \end{aligned}$$

where the computed forward and backward volumes flowing through the considered valve during a cardiac cycle $V_{\text {for}} (\varvec{\phi }_{\textrm{val}})$ and $V_{\text {back}} (\varvec{\phi }_{\textrm{val}})$, respectively, and their measured counterparts $\tilde{V}_{\text {for}}$ and $\tilde{V}_{\text {back}}$ are evaluated by the integration in time $t \in [t_0, t_0+T_{cycl}]$ of the corresponding flow as

40 $$\begin{aligned} V_{\text {for}} (\varvec{\phi }_{\textrm{val}}, t)&= \int _{t_{0}}^{t_{0}+T_{cycl}} |q(\varvec{\phi }_{\textrm{val}}, t) |_{+} \mathrm {~d}t,\end{aligned}$$

41 $$\begin{aligned} \tilde{V}_{\text {for}}(t)&= \int _{t_{0}}^{t_{0}+T_{cycl}} |\tilde{q}(t) |_{+}\mathrm {~d}t, \end{aligned}$$

42 $$\begin{aligned} V_{\text {back}} (\varvec{\phi }_{\textrm{val}}, t)&= \int _{t_{0}}^{t_{0}+T_{cycl}} |q(\varvec{\phi }_{\textrm{val}}, t) |_{-} \mathrm {~d}t, \end{aligned}$$

43 $$\begin{aligned} \tilde{V}_{\text {back}}(t)&= \int _{t_{0}}^{t_{0}+T_{cycl}} |\tilde{q}(t) |_{-}\mathrm {~d}t, \end{aligned}$$

where $|q(t) |_{+}= \text {max}(0,q(t))$ and $|q(t) |_{-}= \text {min}(0,q(t))$, respectively. The computed flow through the valve $q(\varvec{\phi }_{\textrm{val}}, t)$ is evaluated by solving the forward problem described by (9-11), where the pressure gradient $\tilde{p}_{\textrm{v}}^{\ell } - \tilde{p}_{\textrm{ar}}^{\mathrm{{sys}}}$ is taken from the clinical measurements. For the aortic valve model, for example, this yields

44 $$\begin{aligned} \tilde{p}_{\textrm{v}}^{\ell } - \tilde{p}_{\textrm{ar}}^{\mathrm{{sys}}} = \frac{\rho }{2 {A_{\textrm{eff}}^{\mathrm{{aor}}}}^{2}} q_{\textrm{v,out}}^{\ell } |q_{\textrm{v,out}}^{\ell } |+ \frac{\rho l_{\textrm{eff}}^{\mathrm{{aor}}}}{A_{\textrm{eff}}^{\mathrm{{aor}}}} \frac{\textrm{d} q_{\textrm{v,out}}^{\ell }}{\textrm{d}t}, \end{aligned}$$

with

45 $$\begin{aligned} A_{\textrm{eff}}^{\mathrm{{aor}}} = \left[ A_{\textrm{eff,max}}^{\mathrm{{aor}}} - A_{\textrm{eff,min}}^{\mathrm{{aor}}} \right] \ \zeta ^{\mathrm{{aor}}} + A_{\textrm{eff,min}}^{\mathrm{{aor}}}, \end{aligned}$$

and

46 $$\begin{aligned} \frac{\textrm{d} \zeta ^{\mathrm{{aor}}}}{\textrm{d} t}= {\left\{ \begin{array}{ll}(1-\zeta ^{\mathrm{{aor}}}) \ K_{\textrm{v o}}^{\mathrm{{aor}}} \ \Delta \tilde{p}^{\mathrm{{aor}}}, & \Delta \tilde{p}^{\mathrm{{aor}}}> 0, \\ \zeta ^{\mathrm{{aor}}} \ K_{\textrm{v c}}^{\mathrm{{aor}}} \ \Delta \tilde{p}^{\mathrm{{aor}}}, & \Delta \tilde{p}^{\mathrm{{aor}}} \le 0, \end{array}\right. } \end{aligned}$$

where $\Delta \tilde{p}^{\mathrm{{aor}}} = \tilde{p}_{\textrm{v}}^{\ell } - \tilde{p}_{\textrm{ar}}^{\mathrm{{sys}}}$ and $t \in [t_0, t_0+T_{cycl}]$. When the considered valve is closed at $t = t_0$, the start of atrial contraction, the initial conditions read

47 $$\begin{aligned}&q_{\textrm{v,out}}^{\ell }(t_0) = \tilde{q}_{\textrm{v,out}}^{\ell }(t_0) = 0, \end{aligned}$$

48 $$\begin{aligned}&\zeta ^{\mathrm{{aor}}}(t_0) = 0. \end{aligned}$$

The initial conditions can be extracted from the respective clinical measurements. The system of ODEs (44) and (46) is solved for primary variables $\zeta ^{\mathrm{{aor}}}$ and $q_{\textrm{v,out}}^{\ell }$ with a One-Step-$\theta$ time integration Barclay et al (2000). Two separate inverse problems of the form (38) are solved independently for the identification of the maximum and minimum effective orifice area parameters $\varvec{\phi }_{\textrm{val}}^{\mathrm{{pul}}} = \big [ A_{\textrm{eff,max}}^{\mathrm{{pul}}}, A_{\textrm{eff,min}}^{\mathrm{{pul}}} \Big ]$ and $\varvec{\phi }_{\textrm{val}}^{\mathrm{{aor}}} =\big [ A_{\textrm{eff,max}}^{\mathrm{{aor}}}, A_{\textrm{eff,min}}^{\mathrm{{aor}}} \Big ]$ of the pulmonary and aortic valve, respectively.

The effective orifice area parameters of the mitral and tricuspid valves cannot be identified with similar inverse problems, given that time-resolved measurements of atrial pressures and flows through these valves are unavailable. In this case, the maximum geometric orifice area estimated from cine cardiac MRI data is used as the maximum effective orifice area. The minimum effective orifice area can be set to zero when the respective valve exhibits no regurgitation.

For solving 0D inverse subproblems of the form (31) and (38), the L-BFGS optimization algorithm with a cubic line search from the MATLAB Optimization Toolbox is utilized (MATLAB 2022; Broyden 1970; Fletcher 1970; Goldfarb 1970; Shanno 1970).

#### Identification of 3D submodel parameters

The starting times of ventricular contraction $t_{\text {contr,v}}$ and relaxation $t_{\text {relax,v}}$ as well as the starting times of atrial contraction $t_{\text {contr,at}}$ and relaxation $t_{\text {relax,at}}$ are extracted from the electrocardiogram performed during the cine MRI and are required in (19, 20). The active stress parameters are the upstroke rate $\alpha _{\textrm{max}}$, relaxation rate $\alpha _{\textrm{min}}$, and the maximum contractility $\sigma _{0}$. They are assumed to be spatially constant within each heart chamber in our model and govern its respective active stress evolution according to (16, 17). They are identified by solving the minimization problem

49 $$\begin{aligned} \mathop {\textrm{argmin}}\limits _{\varvec{\phi }} f_{\tau }(\varvec{\phi }), \end{aligned}$$

where

50 $$\begin{aligned} \begin{aligned} \varvec{\phi } = \big [&\sigma _{0,\textrm{v}}^{r} \ \alpha _{\textrm{max,v}}^{r} \ \alpha _{\textrm{min,v}}^{r} \ \sigma _{0,\textrm{v}}^{\ell } \ \alpha _{\textrm{max,v}}^{\ell } \ \alpha _{\textrm{min,v}}^{\ell } \\&\sigma _{0,\textrm{at}}^{r} \ \alpha _{\textrm{max,at}}^{r} \ \alpha _{\textrm{min,at}}^{r} \ \sigma _{0,\textrm{at}}^{\ell } \ \alpha _{\textrm{max,at}}^{\ell } \ \alpha _{\textrm{min,at}}^{\ell } \big ]^{T}, \end{aligned} \end{aligned}$$

and

51 $$\begin{aligned} \begin{aligned} f_{\tau }(\varvec{\phi })=&\sum _{i \in \{ r, \ell \} } \Bigg [ \sum _{k \in \mathcal {T}_{\textrm{v}}} \left[ V_{\textrm{v}}^{i} \left( \varvec{d}_{k}(\varvec{\phi }) \right) - \tilde{V}_{\textrm{v}}^{i}\left( t_{k} \right) \right] ^{2} \\&+ \sum _{k \in \mathcal {T}_{\textrm{at}}} \left[ V_{\textrm{at}}^{i} \left( \varvec{d}_{k}(\varvec{\phi }) \right) - \tilde{V}_{\textrm{at}}^{i}\left( t_{k} \right) \right] ^{2} \Bigg ], \end{aligned} \end{aligned}$$

subject to the forward problem (29, A). In (51), the volume of a heart chamber at time step *k* is evaluated based on (26). The sets $\mathcal {T}_{\textrm{v}}$ and $\mathcal {T}_{\textrm{at}}$ contain the time steps at which the gap between measured and computed ventricular and atrial volumes has to be minimized, respectively. The selected time steps must fall within the contraction and relaxation phases of the respective chamber, during which active stress affects the model dynamics and the computed volumes.

The inverse problem (49) is solved with Algorithm 2 based on L-BFGS optimization in combination with an adjoint-based gradient evaluation and cubic polynomials line search (Nocedal and Wright 1999). The adjoint problem formulation required for our dynamic coupled model is presented in appendix B. In line 2 of Algorithm 2, the simultaneous fulfillment of the periodicity criterion (30) and optimization convergence criterion is checked. The periodicity error tolerance is set to $\epsilon _{\textrm{cycl}}=0.04$. The optimization convergence is based here on the objective function value. The threshold $\epsilon _{\textrm{sc}}$??must be selected based on the number of measurement points considered in (51) and the acceptable average error between the measured volume and its computed counterpart. For an average error of 0.5 ml and 18 measurement points, the threshold is set to $\epsilon _{\textrm{sc}}=0.5^{2} \times 18 = 4.5$. In line 8 of Algorithm 2, we trigger a periodicity check and an update of initial conditions when either the optimization convergence criterion is fulfilled or a number $N_{f}$ of forward problem evaluations is exceeded. The parameter $N_{f}$ ensures a regular update of initial conditions in order to avoid unnecessary computational effort for minimizing (51), while initial conditions do not satisfy periodicity. $N_{f}$ is set here to 15 evaluations. Note that updating the initial conditions in each iteration is also counterproductive, as it alters the forward problem and requires reinitializing the L-BFGS Hessian approximation, effectively reducing it to a simple gradient descent algorithm.

**Algorithm 2:**
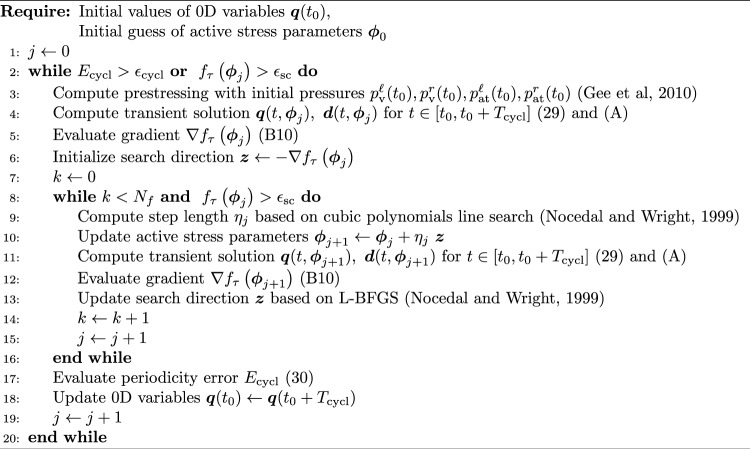
Inverse problem for the identification of active stress parameters

## Results of clinical case study

The inverse analysis framework from Sect. 2.3 is applied to a retrospective repaired ToF patient case to identify patient-specific parameters based on clinical data gathered before a pulmonary valve replacement. The retrospective case serves as a validation experiment to evaluate to which extent the model, calibrated to preoperative data, can predict postoperative states; see also Fig. 2. It is important to note that the postoperative measurements used for the assessment of the outcome prediction are not included in the calibration process.

### Clinical measurements of a repaired ToF patient

The ToF patient (female, 65 kg, 150 cm, 36 y), who underwent surgical repair for ToF as an infant, is considered. Clinical examinations showed a dilated right ventricle with reduced systolic function as well as severe pulmonary regurgitation. The patient underwent pulmonary valve replacement. Preoperative examinations included a cine cardiac MRI, 2D phase-contrast MRI flow measurements through the aorta and main pulmonary artery, and catheterization for pressure measurements, as detailed in Sect. 2.2. The preoperative clinical quantitative integral assessment of ventricular function is given in Table 5.

The patient-specific 3D submodel of the myocardium is shown in Fig. 3. It is segmented from the cine cardiac MRI data of the last phase before atrial contraction, which defines the initial time $t_0$ of the cardiac cycle in the heartbeat simulation depicted in Algorithm 1.

**Table 5:** Preoperative values of the end-diastolic volume (EDV), end-systolic volume (ESV), stroke volume (SV), ejection fraction (EF), end-diastolic volume indexed (EDVI), end-systolic volume indexed (ESVI), forward volume (FV), retrograde volume (RV), and regurgitation fraction (RF) of both ventricles

Ventricle	EDV [$\textrm{ml}$]	ESV [$\textrm{ml}$]	SV [$\textrm{ml}$]	EF [$\mathrm {\%}$]	EDVI [$\frac{\textrm{ml}}{\mathrm {m^{-2}}}$]	ESVI [$\frac{\textrm{ml}}{\mathrm {m^{-2}}}$]	FV [$\textrm{ml}$]	RV [$\textrm{ml}$]	RF [$\mathrm {\%}$]
Right	192.3	116.3	76.0	39.5	118	70	32.9	43.1	56.7
Left	99.7	59.3	40.4	40.5	58	33	39.1	1.3	3.2

**Fig. 5:**
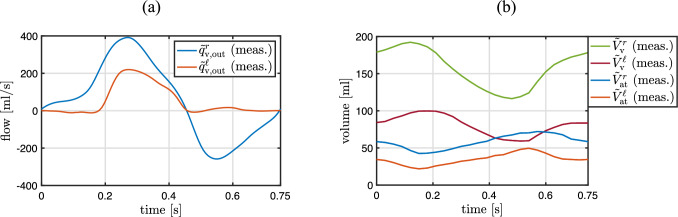
**a** Integral flow measurements through the aortic ($\tilde{q}_{\textrm{v,out}}^{\ell }$) and pulmonary ($\tilde{q}_{\textrm{v,out}}^{r}$) valves based on 2D phase-contrast MRI. **b** Volume measurements of ventricles ($\tilde{V}_{\textrm{v}}^{r}$, $\tilde{V}_{\textrm{v}}^{\ell }$) and atria ($\tilde{V}_{\textrm{at}}^{r}$, $\tilde{V}_{\textrm{at}}^{\ell }$) before pulmonary valve replacement based on cine MRI

**Fig. 6:**
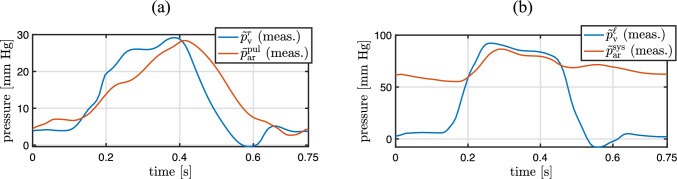
Pressure measurements from catheterization. **a** Right ventricular ($\tilde{p}_{\textrm{v}}^{r}$) and pulmonary arterial ($\tilde{p}_{\textrm{ar}}^{\textrm{pul}}$) pressures. **b** Left ventricular ($\tilde{p}_{\textrm{v}}^{\ell }$) and aortic ($\tilde{p}_{\textrm{ar}}^{\textrm{sys}}$) pressures

MRI phase-contrast integral flow measurements of blood flow through the aortic and pulmonary valves and measured volumes over time of the four heart chambers are given in Fig. 5. Pressure measurements from catheterization in ventricles, aorta, and pulmonary artery are given in Fig. 6.

### Results of the inverse analysis framework

The 3D–0D coupled model of the ToF patient is personalized with the inverse analysis framework presented in Sect. 2.3 and outlined in Table 4 based on the clinical measurements depicted in Sect. 3.1. The values of all personalized and generic 0D and 3D parameters are listed in Tables 7 and 8, respectively.

**Fig. 7:**
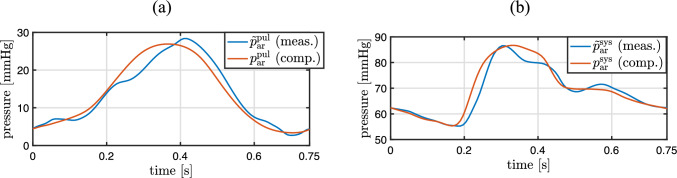
**a** Similarity between measured and computed pulmonary arterial pressure after solving the inverse subproblem (31) for the pulmonary arterial parameters. **b** Similarity between measured and computed systemic arterial pressure after solving the inverse subproblem (31) for the systemic arterial parameters

Patient-specific arterial windkessel parameters of the systemic and pulmonary circulations are computed with two independent inverse subproblems of the form (31). A good agreement between measured and computed arterial pressures is shown in Fig. 7. The computed pressures are the solution of the respective forward subproblem (33) with the identified patient-specific parameter values; see Table 7. The objective functions of both inverse subproblems exhibit a unique minimum. The dependent parameters of the venous windkessel models are derived from their arterial counterparts with the dependency rules (34, 37).

**Fig. 8:**
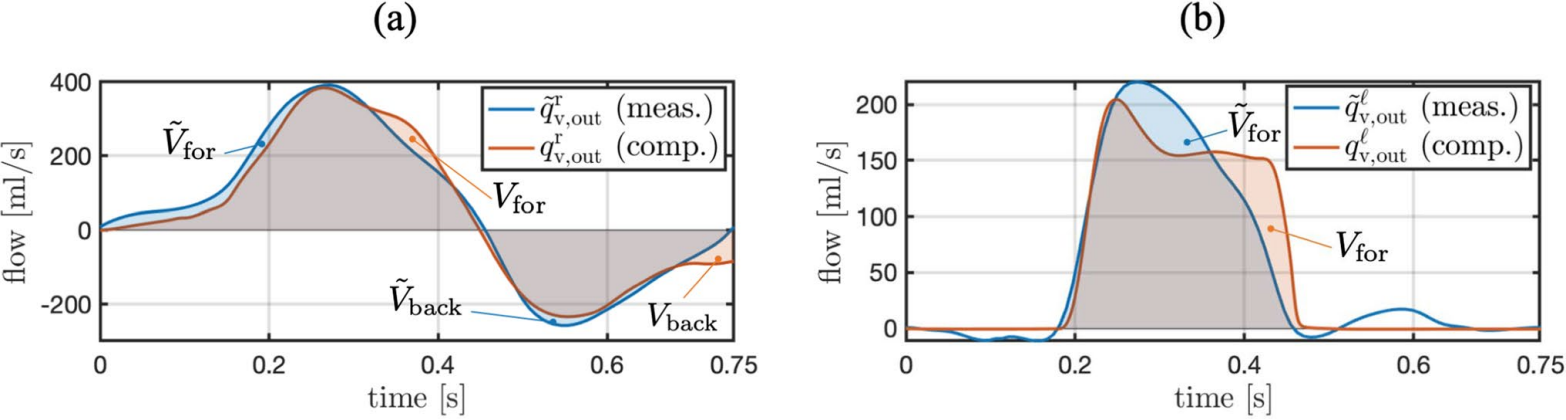
**a** Similarity between measured and computed flow through the pulmonary valve of the ToF patient after solving the inverse subproblem (38). **b** Similarity between measured and computed flow through the aortic valve of the ToF patient after solving the inverse subproblem (38)

The identification of the maximum and minimum effective orifice areas of the aortic and pulmonary valves is performed with two distinct inverse subproblems of the form (38). The identified parameter values are listed in Table 7, and the resulting similarity between measured and computed flow through the aortic and pulmonary valves are outlined in Fig. 8, respectively. The patient-specific parameter values of the mitral and tricuspid valve models are listed in Table 7.

**Fig. 9:**
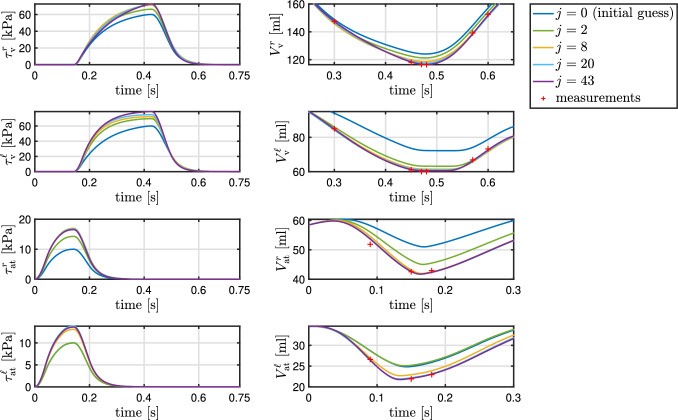
Active stress input functions and corresponding computed volumes of the four heart chambers at different iterations *j* of Algorithm 2 of inverse subproblem (49). The computed volumes converge to the measured values. The resulting active stress parameters are listed in Table 6

**Fig. 10:**
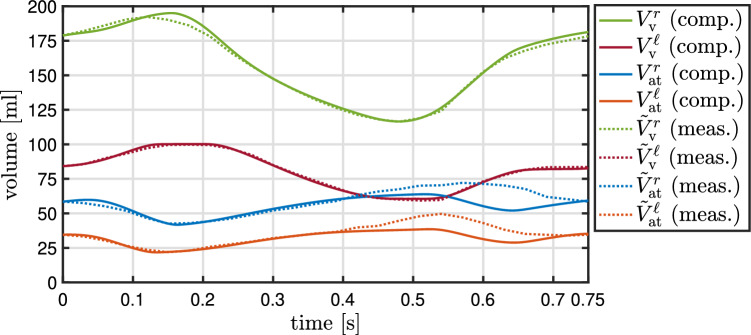
Similarity between measured and computed volumes of the four heart chambers after solving the inverse problem (49) for the active stress parameters

The identification of the active stress parameters of the four heart chambers is performed with the inverse subproblem (49) using Algorithm 2. A comparison between measured volumes of the four heart chambers and their computed counterparts with the identified parameter values is depicted in Fig. 10. The values of the parameters, the objective function, the norm of its gradient, and the periodicity error are depicted in Table 6 for a set of optimization iterations. The solution of the inverse problem required 43 iterations. The active stress functions and the resulting chamber volumes in a set of optimization iterations are illustrated in Fig. 9 for all four heart chambers.

**Table 6:** Convergence of the inverse subproblem (49) for the identification of patient-specific active stress parameters with Algorithm 2. The stopping criterion is the simultaneous fulfillment of the periodicity criterion $E_{\textrm{cycl}} < \epsilon _{\textrm{cycl}}$ and optimization convergence criterion $\ f_{\tau } < \epsilon _{\textrm{sc}}$, where $\epsilon _{\textrm{cycl}}=0.04$ and $\epsilon _{\textrm{sc}}=4.5$

*it*	$\sigma _{0,\textrm{v}}^{r}$	$\alpha _{\textrm{max,v}}^{r}$	$\alpha _{\textrm{min,v}}^{r}$	$\sigma _{0,\textrm{v}}^{\ell }$	$\alpha _{\textrm{max,v}}^{\ell }$	$\alpha _{\textrm{min,v}}^{\ell }$	$\sigma _{0,\textrm{at}}^{r}$	$\alpha _{\textrm{max,at}}^{r}$	$\alpha _{\textrm{min,at}}^{r}$	$\sigma _{0,\textrm{at}}^{\ell }$	$\alpha _{\textrm{max,at}}^{\ell }$	$\alpha _{\textrm{min,at}}^{\ell }$	$f_{\tau }$	$\Vert \nabla f_{\tau } \Vert$	$E_{\textrm{cycl}}$
0	60.0	10.0	$-$30.0	60.0	10.0	$-$30.0	10.0	20.0	$-$30.0	10.0	20.0	$-$30.0	1.079 $\cdot 10^{3}$	1.05 $\cdot 10^{2}$	0.158
2	66.25	11.08	$-$28.72	69.62	15.68	$-$29.30	14.29	20.21	$-$30.04	10.04	20.05	$-$30.03	1.74 $\cdot 10^{2}$	2.43 $\cdot 10^{1}$	0.138
4	69.0	11.02	$-$28.11	71.24	16.55	$-$29.59	15.93	20.34	$-$30.07	11.66	20.11	$-$30.05	7.32 $\cdot 10^{1}$	1.19 $\cdot 10^{1}$	0.131
8	71.23	10.52	$-$27.93	72.29	17.17	$-$30.31	16.99	20.51	$-$30.15	13.10	20.17	$-$30.07	3.47 $\cdot 10^{1}$	4.42	0.126
12	72.05	9.98	$-$28.26	72.67	17.36	$-$30.95	17.27	20.63	$-$30.23	13.69	20.18	$-$30.07	2.68 $\cdot 10^{1}$	2.87	0.123
16	71.92	9.35	$-$29.39	74.62	17.74	$-$31.58	16.83	20.75	$-$30.35	13.68	20.16	$-$30.05	1.65 $\cdot 10^{1}$	1.98	0.033
20	72.20	8.78	$-$30.28	74.95	17.45	$-$32.39	16.78	20.91	$-$30.47	13.82	20.14	$-$30.03	1.33 $\cdot 10^{1}$	1.62	0.034
24	71.84	8.29	$-$31.40	77.49	17.84	$-$32.77	16.96	21.09	$-$30.63	13.39	20.09	$-$29.99	1.15 $\cdot 10^{1}$	2.8	0.026
28	71.64	7.82	$-$32.51	77.75	16.93	$-$33.48	16.66	21.30	$-$30.80	13.57	20.06	$-$29.96	8.35	1.13	0.030
32	71.95	7.59	$-$33.19	78.13	16.29	$-$34.14	16.66	21.51	$-$30.96	13.58	20.02	$-$29.94	6.62	1.10	0.030
36	72.21	7.42	$-$33.71	78.46	15.81	$-$34.75	16.65	21.71	$-$31.11	13.58	19.99	$-$29.91	5.43	9.19 $\cdot 10^{-1}$	0.031
40	72.40	7.28	$-$34.11	78.75	15.43	$-$35.29	16.64	21.91	$-$31.26	13.59	19.95	$-$29.88	4.62	7.70 $\cdot 10^{-1}$	0.032
43	72.55	7.22	$-$34.34	78.92	15.17	$-$35.65	16.54	22.04	$-$31.36	13.61	19.92	$-$29.86	4.18	6.77 $\cdot 10^{-1}$	0.032

**Fig. 11:**
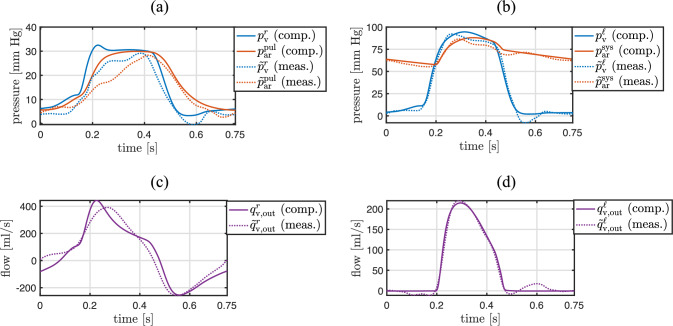
Comparison between measured preoperative quantities and their computed counterparts with the final, calibrated, patient-specific 3D–0D coupled model. **a** Measured and computed pressures in the right ventricle and pulmonary artery. **b** Measured and computed pressures in the left ventricle and aorta. **c** Measured and computed flow through the pulmonary valve, where negative values represent backward flow. **d** Measured and computed flow through the aortic valve

After performing all inverse analysis steps listed in Table 4, pressure and flow quantities computed with the entire patient-specific 3D–0D coupled model also exhibit a good agreement with their measured counterparts, see Fig. 11. This demonstrates that the parameter identifications performed independently using individual submodels are consistent with each other and remain valid within the fully coupled model.

**Fig. 12:**
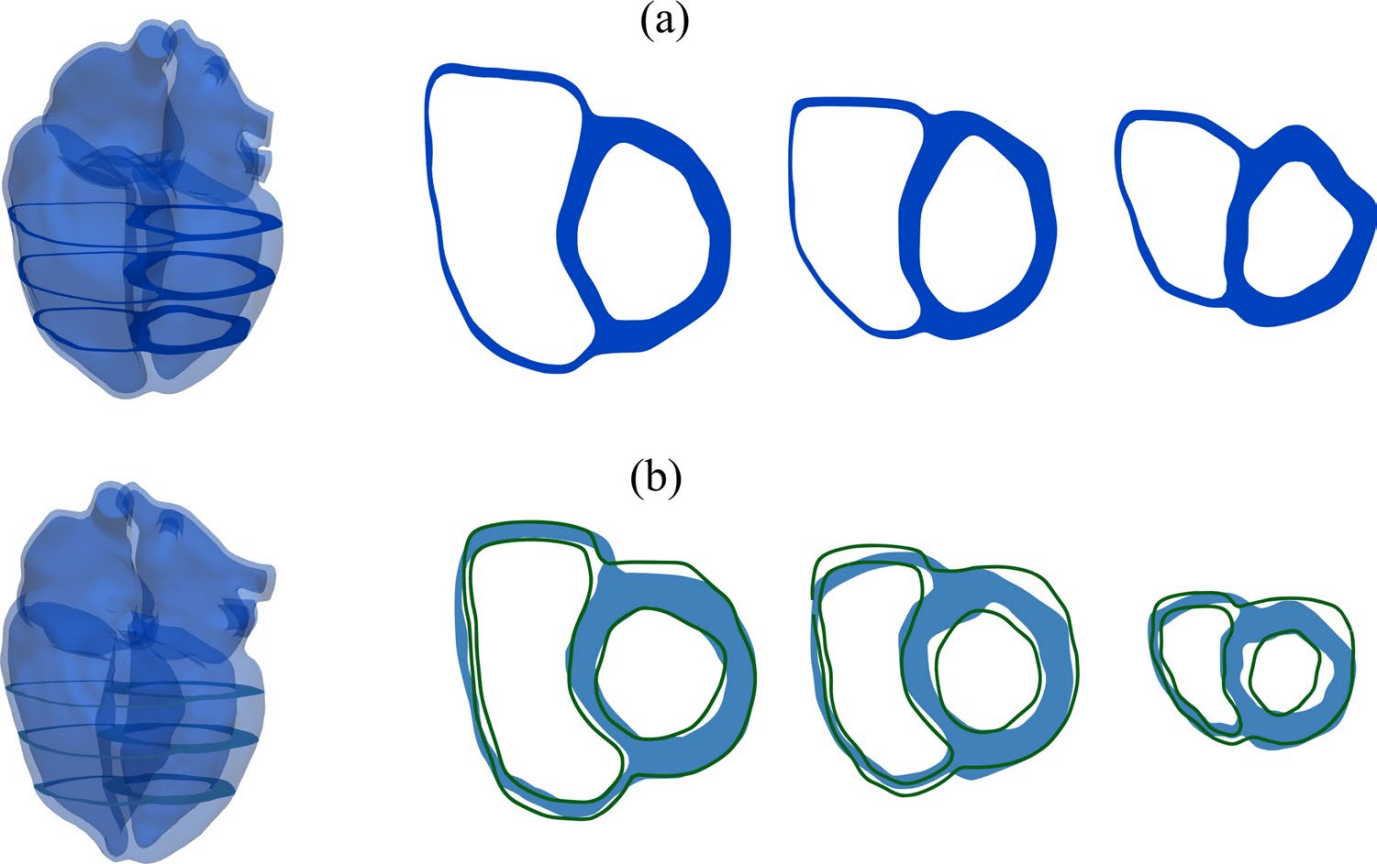
**a** Initial configuration of the heart and slices in short-axis direction at the start of atrial contraction $t = t_0$. **b** Computed end-systolic configuration at $t=t_0 + 0.47$ and slices in short-axis direction (blue) after the parameter identification compared to segmented end-systolic endocardial and epicardial contours (green)

The short-axis views in Fig. 12a show clearly the shift of the septum toward the left side of the heart and the D-shaped left ventricle, which results from the right ventricular dilation and pressure overload. A good agreement between ventricular myocardium’s computed and measured end-systolic configurations can also be observed in Fig. 12b. The short-axis direction is defined from the left ventricular apex point to the center of the aortic valve, and the three illustrated slices are equidistant within this segment. The initial and measured end-systolic configurations are extracted from cine MRI data.

**Table 7:** Values and definition methods of 0D submodel parameters

Submodel	Parameter value	Definition method
Systemic arterial windkessel model	$C_{\textrm{ar}}^{\textrm{sys}}=11302 \ \mathrm {mm^{3} \ kPa^{-1}}$ $R_{\textrm{ar}}^{\textrm{sys}}=1.392 \cdot 10^{-4} \ \mathrm {kPa \ s \ mm^{-3}}$ $Z_{\textrm{ar}}^{\textrm{sys}}=1.308 \cdot 10^{-5} \ \mathrm {kPa \ s \ mm^{-3}}$	Identified with inverse subproblem (31)
Systemic venous windkessel model	$C_{\textrm{ven}}^{\textrm{sys}}=339060 \ \mathrm {mm^{3} \ kPa^{-1}}$ $R_{\textrm{ven}}^{\textrm{sys}}=1.392 \cdot 10^{-5} \ \mathrm {kPa \ s \ mm^{-3}}$	Dependency rule (35) (Gelman et al 2008) Dependency rule (34) (Wang et al 2006; Young 2010)
Pulmonary arterial windkessel model	$C_{\textrm{pul}}^{\textrm{sys}}=19451 \ \mathrm {mm^{3} \ kPa^{-1}}$ $R_{\textrm{pul}}^{\textrm{sys}}=1.33 \cdot 10^{-5} \ \mathrm {kPa \ s \ mm^{-3}}$ $Z_{\textrm{pul}}^{\textrm{sys}}=2.46 \cdot 10^{-6} \ \mathrm {kPa \ s \ mm^{-3}}$	Identified with inverse subproblem (31)
Pulmonary venous windkessel model	$C_{\textrm{ven}}^{\textrm{sys}}=48628 \ \mathrm {mm^{3} \ kPa^{-1}}$ $R_{\textrm{ven}}^{\textrm{sys}}=1.33 \cdot 10^{-5} \ \mathrm {kPa \ s \ mm^{-3}}$	Dependency rule (37) (Tanaka et al 1986) Dependency rule (36) (Gaar Ka et al 1967)
Aortic valve model	$A_{\textrm{eff,max}}^{\textrm{aor}} =1.41 \ \mathrm {cm^{2}}$ $A_{\textrm{eff,min}}^{\textrm{aor}}=2.5 \cdot 10^{-3} \ \mathrm {cm^{2}}$	Identified with inverse subproblem (38)
	$K_{\textrm{v o}}^{\textrm{aor}} =120 \ \mathrm {s^{-1} \ kPa^{-1}}$ $K_{\textrm{v c}}^{\textrm{aor}} =120 \ \mathrm {s^{-1} \ kPa^{-1}}$	Generic values (Mynard et al 2012)
	$l_{\textrm{eff}}^{\textrm{aor}}=39 \ \textrm{mm}$	Dependency rule: 3 times EOA diameter (Flachskampf et al 1993)
	$\rho =10^{-6} \ \mathrm {kg \ mm^{-3}}$	Generic value (Vitello et al 2015)
Pulmonary valve model	$A_{\textrm{eff,max}}^{\textrm{pul}} =3.08 \ \mathrm {cm^{2}}$ $A_{\textrm{eff,min}}^{\textrm{pul}}=1.64 \ \mathrm {cm^{2}}$	Identified with inverse subproblem (38)
	$K_{\textrm{v o}}^{\textrm{pul}} =200 \ \mathrm {s^{-1} \ kPa^{-1}}$ $K_{\textrm{v c}}^{\textrm{pul}} =200 \ \mathrm {s^{-1} \ kPa^{-1}}$	Generic values (Mynard et al 2012)
	$l_{\textrm{eff}}^{\textrm{pul}}=61 \ \textrm{mm}$	Dependency rule: 3 times EOA diameter (Flachskampf et al 1993)
Mitral valve model	$A_{\textrm{eff,max}}^{\textrm{mit}} =2.88 \ \mathrm {cm^{2}}$ $A_{\textrm{eff,min}}^{\textrm{mit}}=1.0 \cdot 10^{-3} \ \mathrm {cm^{2}}$	Identified from cine cardiac MRI data
	$K_{\textrm{v o}}^{\textrm{mit}} =300 \ \mathrm {s^{-1} \ kPa^{-1}}$ $K_{\textrm{v c}}^{\textrm{mit}} =400 \ \mathrm {s^{-1} \ kPa^{-1}}$	Generic values (Mynard et al 2012)
	$l_{\textrm{eff}}^{\textrm{mit}}=54 \ \textrm{mm}$	Dependency rule: 3 times EOA diameter (Flachskampf et al 1993)
Tricuspid valve model	$A_{\textrm{eff,max}}^{\textrm{tri}} =3.46 \ \mathrm {cm^{2}}$ $A_{\textrm{eff,min}}^{\textrm{tri}}=1.0 \cdot 10^{-3} \ \mathrm {cm^{2}}$	Identified from cine cardiac MRI data
	$K_{\textrm{v o}}^{\textrm{tri}} =300 \ \mathrm {s^{-1} \ kPa^{-1}}$ $K_{\textrm{v c}}^{\textrm{tri}} =400 \ \mathrm {s^{-1} \ kPa^{-1}}$	Generic values (Mynard et al 2012)
	$l_{\textrm{eff}}^{\textrm{tri}}=63 \ \textrm{mm}$	Dependency rule: 3 times EOA diameter (Flachskampf et al 1993)

**Table 8:** Values and definition methods of 3D submodel parameters

Submodel	Parameter value		Definition method
Ventricular active stress model	$\sigma _{0,\textrm{v}}^{\ell } =78.92 \ \textrm{kPa}$ $\alpha _{\textrm{max,v}}^{\ell }= 15.17 \ \textrm{s}^{-1}$ $\alpha _{\textrm{min,v}}^{\ell }=-35.65 \ \textrm{s}^{-1}$	$\sigma _{0,\textrm{v}}^{r} = 72.55 \ \textrm{kPa}$ $\alpha _{\textrm{max,v}}^{r}= 7.21 \ \textrm{s}^{-1}$ $\alpha _{\textrm{min,v}}^{r}=-34.34 \ \textrm{s}^{-1}$	Identified with inverse subproblem (51)
	$t_{\textrm{contr,v}}=t_0 + 0.1425 \ \textrm{s}$ $t_{\textrm{relax,v}}=t_0 + 0.4275 \ \textrm{s}$	$T_{\textrm{cycl}}= 0.75 \ \textrm{s}$	Identified from ECG data
Atrial active stress model	$\sigma _{0,\textrm{at}}^{\ell } =13.60 \ \textrm{kPa}$ $\alpha _{\textrm{max,at}}^{\ell }= 19.92 \ \textrm{s}^{-1}$ $\alpha _{\textrm{min,at}}^{\ell }=-29.85 \ \textrm{s}^{-1}$	$\sigma _{0,\textrm{at}}^{r} = 16.54 \ \textrm{kPa}$ $\alpha _{\textrm{max,at}}^{r}= 22.04 \ \textrm{s}^{-1}$ $\alpha _{\textrm{min,at}}^{r}=-31.36 \ \textrm{s}^{-1}$	Identified with inverse subproblem (51)
	$t_{\textrm{contr,at}}=t_0 \ \textrm{s}$ $t_{\textrm{relax,at}}=t_0 + 0.1425 \ \textrm{s}$	$T_{\textrm{cycl}}= 0.75 \ \textrm{s}$	Identified from ECG data
Passive myocardial material model	$a =0.059 \ \textrm{kPa}$ $a_f =18.472 \ \textrm{kPa}$ $a_s =2.481 \ \textrm{kPa}$ $a_{f s}=0.216 \ \textrm{kPa}$	$b = 8.023$ $b_f = 16.026$ $b_s = 11.120$ $b_{f s} = 11.436$	Generic values (Holzapfel and Ogden 2009)
	$\rho _0= 1.053 \cdot 10^{-6} \ \mathrm {kg \ mm^{-3}}$		Generic value (Vinnakota and Bassingthwaighte 2004)
	$\kappa =10^{3} \ \textrm{kPa}$		Chosen large for near-incompressibility
	$c_{K}= 10^{-4} \ \textrm{s}$	$c_{M}= 0.0 \ \mathrm {s^{-1}}$	Generic values (Hirschvogel et al 2017)
Viscoelastic boundary conditions	$k_{\textrm{e}}=0.2 \ \mathrm {kPa \ mm^{-1}}$ $c_{\textrm{e}} =0.005 \ \mathrm {kPa \ s \ mm^{-1}}$	$k_{\textrm{ves}} =2000 \ \mathrm {kPa \ mm^{-1}}$ $c_{\textrm{ves}} =0.01 \ \mathrm {kPa \ s \ mm^{-1}}$	Generic values (Pfaller et al 2019)

### Outcome prediction of a pulmonary valve replacement

The pulmonary valve replacement of the ToF patient is modeled by replacing the value of the minimum effective orifice area of the pulmonary valve model $A_{\textrm{eff,min}}^{\mathrm{{pul}}}$ from the identified, patient-specific preoperative value of $1.64 \ \mathrm {cm^{2}}$ to $0 \ \mathrm {cm^{2}}$, which represents the non-leaky behavior of the implanted artificial valve. All other parameters are left unaltered. The forward simulation described in Algorithm 1 is performed until a periodic postoperative state is reached.

**Fig. 13:**
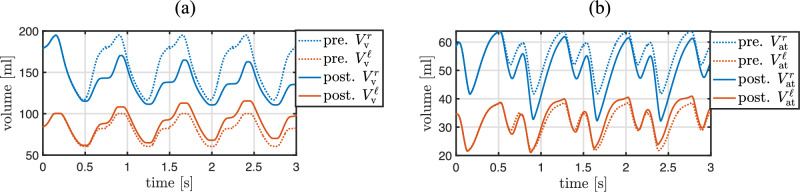
Transient evolution of in silico postoperative ventricular **a** and atrial **b** volumes during the first 4 cardiac cycles after in silico pulmonary valve replacement. The preoperative periodic evolution of the chamber volumes (dashed) is given for comparison

Figure 13a shows the predicted decrease of right ventricular volume and increase of left ventricular volume during the first four heartbeats after the in silico pulmonary valve replacement. The atrial volumes also exhibit similar behavior as depicted in Fig. 13b. The cardiovascular model converges to a new periodic postoperative state.

**Fig. 14:**
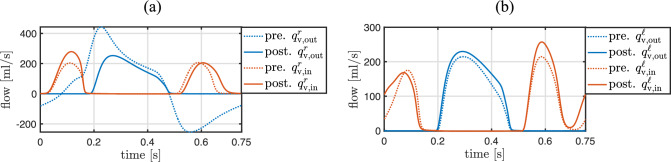
**a** Comparison between in silico preoperative (dashed) and predicted postoperative flow through the replaced pulmonary valve and the tricuspid valve. **b** Comparison between in silico preoperative (dashed) and predicted postoperative flow through the aortic and mitral valves

Figure 14a shows the elimination of pulmonary regurgitation and an increase of flow through the tricuspid valve during diastole. Although the forward pulmonary flow decreased during systole, the net forward pulmonary flow in a cardiac cycle increased, as there is no backward flow during diastole anymore. Figure 14b shows increased flow through the aortic and mitral valves. This indicates that pulmonary valve replacement led to an increase in cardiac output.

**Fig. 15:**
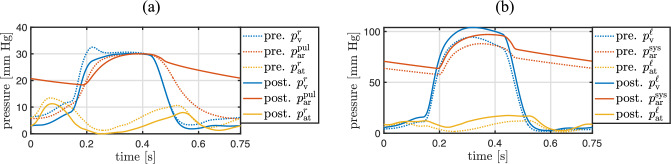
**a** Comparison between in silico preoperative (dashed) and predicted postoperative pressures in the right ventricle, right atrium, and pulmonary artery. **b** Comparison between in silico preoperative (dashed) and predicted postoperative pressures in the left ventricle, left atrium, and aorta

Figure 15a and b show the in silico predicted postoperative ventricular, arterial, and atrial pressures in comparison with their preoperative counterparts in the right and left side of the heart, respectively. A significant increase in the diastolic pressure gradient through the pulmonary valve and a decrease in right ventricular and atrial pressures can be observed. Conversely, left ventricular, atrial, and aortic pressures have increased due to the rise in cardiac output and the higher net forward flow ejected from the right side of the heart.

**Fig. 16:**
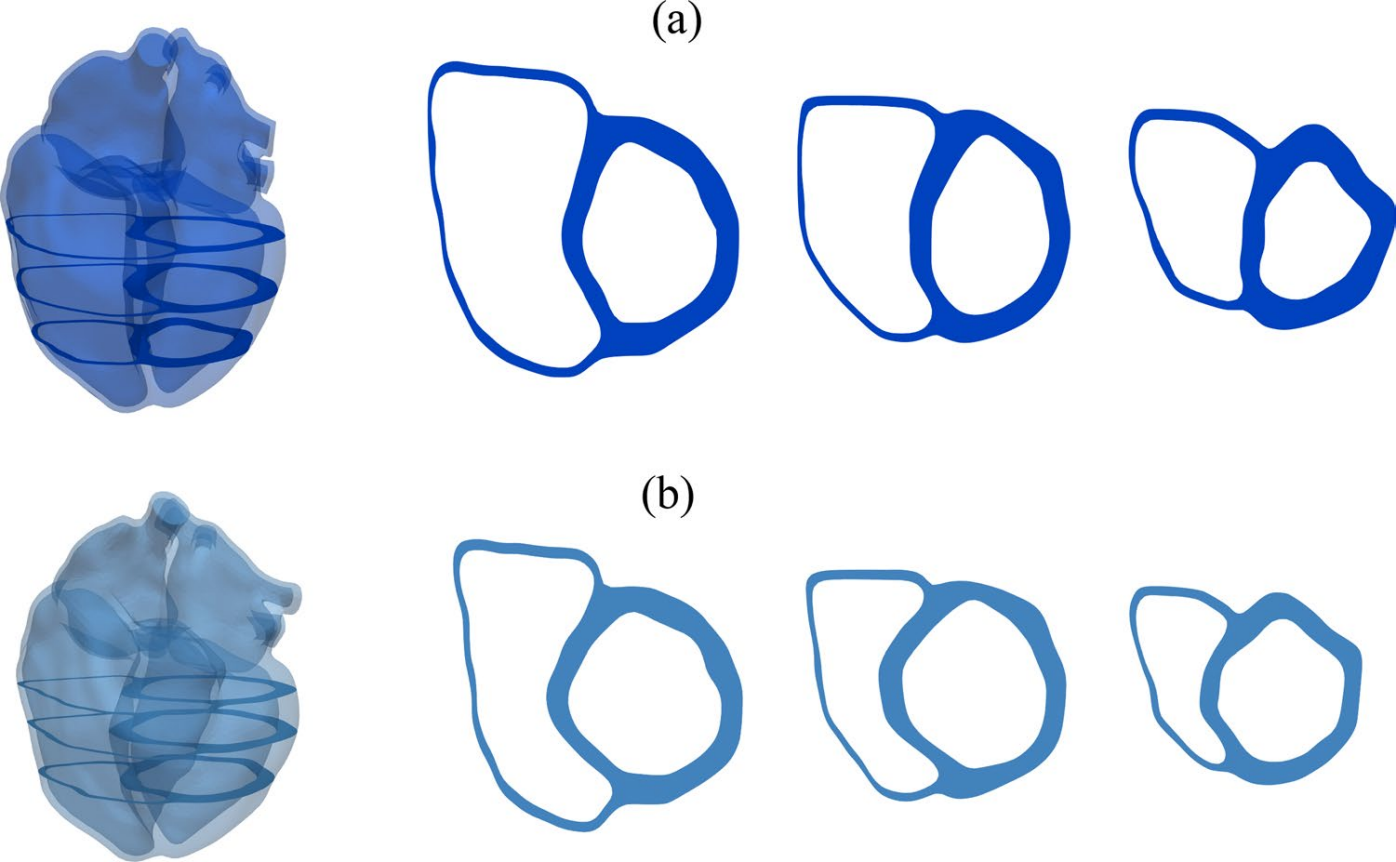
**a** Preoperative configuration of the heart muscle and slices in short-axis direction at the start of atrial contraction. **b** In silico predicted postoperative configuration of the heart muscle and slices in short-axis direction at the start of atrial contraction

Figure 16a shows how the right ventricular dilation in the preoperative state shifts the interventricular septum toward the left, changing left ventricular geometry to a D-shaped form. The postoperative prediction in Figure 16b demonstrates the instantaneous effect of pulmonary valve replacement in changing the left ventricular form to a normal shape. The decrease in right ventricular volume observed in the short-axis views is consistent with the volume curves presented in Fig. 13a.

### Prediction assessment

A postoperative cardiac MRI examination of the patient was performed 15 months after the in vivo pulmonary valve replacement to validate the success of the intervention quantitatively. Pressure measurements using catheterization were not performed after the valve replacement. It is important to note that a 15-month period between valve replacement and postoperative measurements is sufficient for reverse remodeling and growth processes to take effect, potentially influencing short-term cardiac mechanics. However, the patient-specific computational model in this study accounts only for the immediate effects of valve replacement post-intervention and does not incorporate long-term phenomena such as growth and remodeling. Therefore, the conducted postoperative measurements can only be used as a qualitative reference for expected postoperative changes and do not allow for a quantitative assessment of the computational predictions on a short-time scale.

The comparison of measured and predicted postoperative quantities with preoperative quantities depicted in Table 9 shows that all predicted changes in the right ventricular function are qualitatively in agreement with the measurements and are physiologically meaningful. In the predicted postoperative simulation, the right ventricular end-diastolic, end-systolic, and stroke volumes decreased significantly. The change of all key quantities occurred in the expected, physiologically consistent direction. The end-diastolic right ventricular volume decreased, for instance, by 31.2 ml in the predictions, compared to a decrease of 58.3 ml according to the 15-month delayed measurements. On the other hand, the decrease of right ventricular stroke volume was accurately predicted to fall from a preoperative value of 76 ml to a postoperative value of 47.2 ml compared to the postoperative measurement of 49.1 ml. This difference can be explained by the reverse growth and remodeling processes during the 15 months between the valve replacement and the measurements. The results in Table 9 suggest that the predicted short-time scale changes directly after the pulmonary valve replacement trigger a long time scale reverse growth and remodeling such that the right ventricular dilation is reduced.

**Fig. 17:**
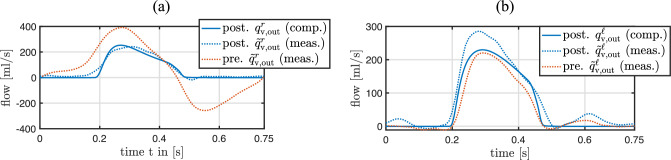
**a** Comparison between preoperative and postoperative measurements of flow through the replaced pulmonary valve and the computed postoperative prediction. **b** Comparison between preoperative and postoperative measurements of flow through the aortic valve and the computed postoperative prediction

The predicted flow through the pulmonary valve in Fig. 17a is in good agreement with the respective postoperative measurement. Although the predicted aortic flow in 17b increased as expected, it exhibits a minor increase compared to the respective postoperative measurement.

**Table 9:** Quantitative comparison between preoperative, predicted postoperative and measured postoperative values of the end-diastolic volume (EDV), end-systolic volume (ESV), stroke volume (SV), ejection fraction (EF), end-diastolic volume indexed (EDVI), end-systolic volume indexed (ESVI), forward volume (FV), retrograde volume (RV), and regurgitation fraction (RF) of both ventricles

Ventricle	EDV [$\textrm{ml}$]	ESV [$\textrm{ml}$]	SV [$\textrm{ml}$]	EF [$\mathrm {\%}$]	FV [$\textrm{ml}$]	RV [$\textrm{ml}$]	RF [$\mathrm {\%}$]
*Preoperative measurements*							
Right	192.3	116.3	76.0	39.5	32.9	43.1	56.7
Left	99.7	59.3	40.4	40.5	39.1	1.3	3.2
*Postoperative predictions*							
Right	161.1	113.9	47.2	29.3	47.0	0.2	0.42
Left	120.2	74.4	45.8	38.1	45.6	0.2	0.44
*Postoperative measurements*							
Right	134	84.9	49.1	37.3	48.1	1	2
Left	105.5	50.5	55	52.1	52	3	5.4

**Fig. 18:**
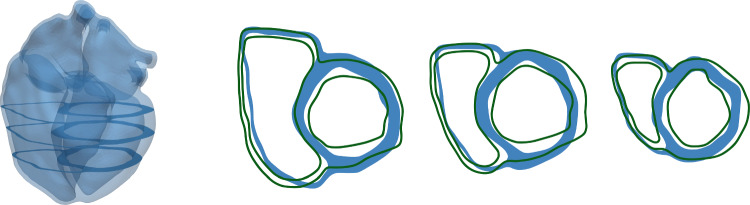
Predicted postoperative configuration of the heart muscle and slices in short-axis direction (blue) at the start of atrial contraction $t=t_0$ compared to measured postoperative endocardial and epicardial contours (green), segmented from cine MRI data

## Discussion

The proposed decomposition of the global parameter identification problem into independent inverse subproblems is more efficient and robust than solving simultaneously for all 3D and 0D parameters with the entire 3D–0D model as the forward problem. The formulated inverse subproblems are identifiable and exhibit a unique solution, which satisfies all prescribed similarities between computed and measured target quantities. This decomposition is feasible due to the availability of clinical data at the interfaces between submodels, which are prescribed during the independent solution of submodels. It avoids propagation of identification errors, given that submodels do not rely on computed coupling quantities from neighboring submodels to evaluate their response.

With the proposed approach, we were able to compute personalized parameters of a high-fidelity model with manageable computational effort and time for clinical practice. The calibrated model is able to represent the measured patient-specific responses; see Figs. 11 and 12. The pulmonary valve replacement predictions in Sect. 3.3 are physiologically consistent and correlate well with postoperative measurements, as shown in Table 9 and Figs. 17 and 18. Although most of the key quantities evolved in a physiologically meaningful direction, the extent of the change was not accurately predicted in some quantities. The causes of this discrepancy will be discussed in the limitations section below.

While cine cardiac MRI data allow for the extraction of endocardial and epicardial surfaces throughout the cardiac cycle, the segmented surfaces lack kinematic information, such as node-to-node correspondence, necessary for uniquely identifying local material properties. Therefore, we assume a spatially uniform active stress value within each chamber and use volume measurements of the heart chambers as target quantities for identifying active stress parameters.

To successfully implement the proposed approach, detecting, correcting, or removing inaccurate or inconsistent measurements with the guidance of experienced clinicians is required. Cine MRI volume measurements and 2D phase-contrast MRI flow measurements were not completely consistent with each other and did not exactly fulfill the conservation of mass. Cardiac radiology experts suggested eliminating the inconsistency by relying on flow measurements and adjusting volume measurements.

Heart rate variations cause slight differences in cardiac cycle durations in different measurements. They are corrected by scaling the time axis of all measurements to match the heart rate in the flow measurement. Similar correction strategies were adopted by Schiavazzi et al (2017). The heart rate variations are minor enough to assume the same ratios of systolic and diastolic durations. Therefore, the cardiac cycle durations are scaled uniformly over all phases. The time axis of all measurements is also shifted, such that the initial time $t_0$ coincides with the beginning of atrial contraction, which corresponds to the P wave on the ECG.

## Limitations

Figure 10 shows a higher discrepancy between measured and computed atrial volumes during passive filling and emptying. A higher discrepancy between measured and computed quantities is also observed in the right side of the heart when compared to the left side, as shown in Fig. 11. The atria and right ventricle have a thinner wall and lower pressures than the left ventricle. They are, therefore, more sensitive to viscoelastic boundary conditions which model the interaction forces with the pericardium. This sensitivity is more pronounced during the passive emptying and filling phases. For this reason, achieving accurate patient-specific model responses in thin-walled, low-pressure chambers is more challenging given that the optimization parameters to be identified include only active stress parameters.

With the currently available data, viscoelastic properties of the pericardial interaction and passive myocardial parameters cannot be individually identified. In future work, and with the availability of more detailed measurements, the identification of personalized viscoelastic parameters for the pericardial interaction model, as well as the passive myocardial tissue properties, will be important for achieving a more accurate patient-specific response and improving the reliability of model predictions.

Additionally, the model assumes spatially homogeneous activation for each chamber. The delay associated with the propagation of action potentials through the myocardial syncytium is not represented, which may limit the accuracy of simulations where spatial and temporal patterns of myocardial activation play a significant role.

Another modeling limitation is the absence of valve leaflets in the geometric model. This simplification stems from the use of a 0D lumped-parameter flow model, which does not resolve spatial fluid dynamics or enable fluid–structure interaction. While valve behavior is modeled through ODEs governing flow and pressure relationships, the lack of geometrically represented leaflets prevents the analysis of local hemodynamics, mechanical stresses, and deformation at the valve level. This may affect the interpretation of results in contexts where leaflet dynamics are physiologically relevant.

The model parameters are identified based on preoperative measurements and are assumed to remain constant. As such, the current model does not account for the variability of parameters that may arise from long-term physiological processes such as growth and remodeling. Consequently, the model is intended to represent patient-specific behavior in the short-term, and its predictive ability may be limited for long-term scenarios without recalibration or the inclusion of remodeling mechanisms.

The non-simultaneous measurement of ventricular and arterial pressures makes the estimation of pressure gradients through semilunar valves complex and induces measurement errors.

Flows through the atrioventricular valves were not measured for the patient under consideration. Consequently, the corresponding submodels cannot be calibrated using inverse analysis, as performed for the semilunar valves. Instead, their maximum and minimum effective orifice areas were directly estimated from MRI data based on the geometric orifice area. If atrial and ventricular pressures, along with the flow through an atrioventricular valve, are available, its parameters can be identified by solving an inverse problem of the form (38).

The catheter measurements in chambers and arteries with lower pressures, such as the right ventricle and pulmonary artery, exhibit more significant relative errors and measurement uncertainty, which might increase the uncertainty of inverse analysis results of pulmonary valve and pulmonary arteries model parameters.

## Figures and Tables

**Fig. 19:**
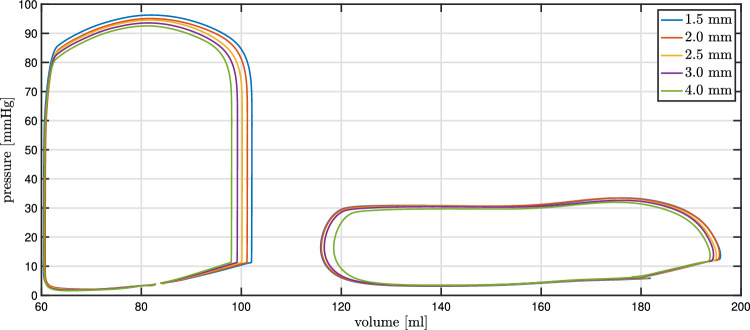
Pressure volume loops of left and right ventricles for different average element sizes

## Data Availability

No datasets were generated or analyzed during the current study.

## Time discretized 0D equations

The residual vector of the 0D submodels of the vascular system and heart valves is discretized with the One-Step-$\theta$ scheme Barclay et al (2000) and reads

$$\begin{aligned} \begin{aligned}&\varvec{R}^{\textrm{0D}}_{k+1} = \\ &\begin{bmatrix} \frac{V_{\textrm{at}}^{\ell }\left( \varvec{d}_{k+1}\right) -V_{\textrm{at}}^{\ell }\left( \varvec{d}_k\right) }{\Delta t}-q_{\textrm{ven}, k+\theta }^{\textrm{pul}}+q_{\textrm{v}, \textrm{in}, k+\theta }^{\ell } \\ \frac{q_{\textrm{v,in},k+1}^{\ell } - q_{\textrm{v,in},k}^{\ell }}{\Delta t} + \theta \frac{ \rho \ q_{\textrm{v,in},k+1}^{\ell } |q_{\textrm{v,in},k+1}^{\ell } |- 2 \left( p_{\textrm{at},k+1}^{\ell } - p_{\textrm{v},k+1}^{\ell }\right) \left[ \left( A_{\textrm{eff,max}}^{\textrm{mit}} - A_{\textrm{eff,min}}^{{\textrm{mit}}} \right) \ \zeta _{k+1}^{{\textrm{mit}}} + A_{\textrm{eff,min}}^{{\textrm{mit}}}\right] ^{2} }{2 \rho \ l_{\textrm{eff}}^{{\textrm{mit}}} \left[ \left( A_{\textrm{eff,max}}^{{\textrm{mit}}} - A_{\textrm{eff,min}}^{{\textrm{mit}}} \right) \ \zeta _{k+1}^{{\textrm{mit}}} + A_{\textrm{eff,min}}^{{\textrm{mit}}}\right] } + (1-\theta ) \frac{ \rho \ q_{\textrm{v,in},k}^{\ell } |q_{\textrm{v,in},k}^{\ell } |- 2 \left( p_{\textrm{at},k}^{\ell } - p_{\textrm{v},k}^{\ell }\right) \left[ \left( A_{\textrm{eff,max}}^{{\textrm{mit}}} - A_{\textrm{eff,min}}^{{\textrm{mit}}} \right) \ \zeta _{k}^{{\textrm{mit}}} + A_{\textrm{eff,min}}^{{\textrm{mit}}}\right] ^{2} }{2 \rho \ l_{\textrm{eff}}^{{\textrm{mit}}} \left[ \left( A_{\textrm{eff,max}}^{{\textrm{mit}}} - A_{\textrm{eff,min}}^{{\textrm{mit}}} \right) \ \zeta _{k}^{{\textrm{mit}}} + A_{\textrm{eff,min}}^{{\textrm{mit}}}\right] } \\ { {\left\{ \begin{array}{ll} \frac{\zeta ^{{\textrm{mit}}}_{k+1} - \zeta ^{{\textrm{mit}}}_{k}}{\Delta t} - \theta (1-\zeta ^{{\textrm{mit}}}_{k+1}) \ K_{\textrm{v o}}^{{\textrm{mit}}} \left( p_{\textrm{at},k+1}^{\ell } - p_{\textrm{v},k+1}^{\ell }\right) - (1-\theta ) (1-\zeta ^{{\textrm{mit}}}_{k}) \ K_{\textrm{v o}}^{{\textrm{mit}}} \left( p_{\textrm{at},k}^{\ell } - p_{\textrm{v},k}^{\ell }\right) , \quad \quad & \text {if} \quad p_{\textrm{at},k+1}^{\ell } - p_{\textrm{v},k+1}^{\ell }>0, \\ \frac{\zeta ^{{\textrm{mit}}}_{k+1} - \zeta ^{{\textrm{mit}}}_{k}}{\Delta t} - \theta \zeta ^{{\textrm{mit}}}_{k+1} \ K_{\textrm{v c}}^{{\textrm{mit}}} \ \left( p_{\textrm{at},k+1}^{\ell } - p_{\textrm{v},k+1}^{\ell }\right) - (1-\theta ) \zeta ^{{\textrm{mit}}}_{k} \ K_{\textrm{v c}}^{{\textrm{mit}}} \ \left( p_{\textrm{at},k}^{\ell } - p_{\textrm{v},k}^{\ell }\right) , \quad \quad & \text {if} \quad p_{\textrm{at},k+1}^{\ell } - p_{\textrm{v},k+1}^{\ell } \le 0,\end{array}\right. }} \\ \frac{V_{\textrm{v}}^{\ell }\left( \varvec{d}_{k+1}\right) -V_{\textrm{v}}^{\ell }\left( \varvec{d}_k\right) }{\Delta t}-q_{\textrm{v,in}, k+\theta }^{\ell }+q_{\textrm{v,out}, k+\theta }^{\ell }\\ \frac{q_{\textrm{v,out}, k+1}^{\ell }-q_{\textrm{v,out}, k}^{\ell }}{\Delta t} + \theta \frac{ \rho \ q_{\textrm{v,out},k+1}^{\ell } |q_{\textrm{v,out},k+1}^{\ell } |- 2 \left( p_{\textrm{v},k+1}^{\ell } - p_{\textrm{ar},k+1}^{{\textrm{sys}}}\right) \left[ \left( A_{\textrm{eff,max}}^{{\textrm{aor}}} - A_{\textrm{eff,min}}^{{\textrm{aor}}} \right) \ \zeta _{k+1}^{{\textrm{aor}}} + A_{\textrm{eff,min}}^{{\textrm{aor}}}\right] ^{2} }{2 \rho \ l_{\textrm{eff}}^{{\textrm{aor}}} \left[ \left( A_{\textrm{eff,max}}^{{\textrm{aor}}} - A_{\textrm{eff,min}}^{{\textrm{aor}}} \right) \ \zeta _{k+1}^{{\textrm{aor}}} + A_{\textrm{eff,min}}^{{\textrm{aor}}}\right] } + (1-\theta ) \frac{ \rho \ q_{\textrm{v,out},k}^{\ell } |q_{\textrm{v,out},k}^{\ell } |- 2 \left( p_{\textrm{v},k}^{\ell } - p_{\textrm{ar},k}^{{\textrm{sys}}}\right) \left[ \left( A_{\textrm{eff,max}}^{{\textrm{aor}}} - A_{\textrm{eff,min}}^{{\textrm{aor}}} \right) \ \zeta _{k}^{{\textrm{aor}}} + A_{\textrm{eff,min}}^{{\textrm{aor}}}\right] ^{2} }{2 \rho \ l_{\textrm{eff}}^{{\textrm{aor}}} \left[ \left( A_{\textrm{eff,max}}^{{\textrm{aor}}} - A_{\textrm{eff,min}}^{{\textrm{aor}}} \right) \ \zeta _{k}^{{\textrm{aor}}} + A_{\textrm{eff,min}}^{{\textrm{aor}}}\right] } \\ { {\left\{ \begin{array}{ll} \frac{\zeta ^{{\textrm{aor}}}_{k+1} - \zeta ^{{\textrm{aor}}}_{k}}{\Delta t} - \theta (1-\zeta ^{{\textrm{aor}}}_{k+1}) \ K_{\textrm{v o}}^{{\textrm{aor}}} \left( p_{\textrm{v},k+1}^{\ell } - p_{\textrm{ar},k+1}^{{\textrm{sys}}}\right) - (1-\theta ) (1-\zeta ^{{\textrm{aor}}}_{k}) \ K_{\textrm{v o}}^{{\textrm{aor}}} \left( p_{\textrm{v},k}^{\ell } - p_{\textrm{ar},k}^{{\textrm{sys}}}\right) , \quad \quad & \text {if} \quad p_{\textrm{v},k+1}^{\ell } - p_{\textrm{ar},k+1}^{{\textrm{sys}}}>0, \\ \frac{\zeta ^{{\textrm{aor}}}_{k+1} - \zeta ^{{\textrm{aor}}}_{k}}{\Delta t} - \theta \zeta ^{{\textrm{aor}}}_{k+1} \ K_{\textrm{v c}}^{{\textrm{aor}}} \ \left( p_{\textrm{v},k+1}^{\ell } - p_{\textrm{ar},k+1}^{{\textrm{sys}}}\right) - (1-\theta ) \zeta ^{{\textrm{aor}}}_{k} \ K_{\textrm{v c}}^{{\textrm{aor}}} \ \left( p_{\textrm{v},k}^{\ell } - p_{\textrm{ar},k}^{{\textrm{sys}}}\right) , \quad \quad & \text {if} \quad p_{\textrm{v},k+1}^{\ell } - p_{\textrm{ar},k+1}^{{\textrm{sys}}} \le 0,\end{array}\right. }} \\ C_{\textrm{ar}}^{{\textrm{sys}}}\left( \frac{p_{\textrm{ar}, k+1}^{{\textrm{sys}}}-p_{\textrm{ar}, k}^{{\textrm{sys}}}}{\Delta t}-Z_{\textrm{ar}}^{{\textrm{sys}}} \frac{q_{\textrm{v,out}, k+1}^{\ell }-q_{\textrm{v,out}, k}^{\ell }}{\Delta t}\right) -q_{\textrm{v,out}, k+\theta }^{\ell }+q_{\textrm{ar}, k+\theta }^{{\textrm{sys}}} \\ \frac{1}{R_{\textrm{ar}}^{{\textrm{sys}}}} \left( p_{\textrm{ven}, k+\theta }^{{\textrm{sys}}} - p_{\textrm{ar}, k+\theta }^{{\textrm{sys}}} + Z_{\textrm{ar}}^{{\textrm{sys}}} q_{\textrm{v,out}, k+\theta }^{\ell }\right) + q_{\textrm{ar}, k+\theta }^{{\textrm{sys}}} \\ C_{\textrm{ven}}^{{\textrm{sys}}}\frac{p_{\textrm{ven}, k+1}^{{\textrm{sys}}}-p_{\textrm{ven}, k}^{{\textrm{sys}}}}{\Delta t}-q_{\textrm{ar}, k+\theta }^{{\textrm{sys}}} + q_{\textrm{ven}, k+\theta }^{{\textrm{sys}}}\\ \frac{1}{R_{\textrm{ven}}^{{\textrm{sys}}}} \left( p_{\textrm{at}, k+\theta }^{r} - p_{\textrm{ven}, k+\theta }^{{\textrm{sys}}} \right) + q_{\textrm{ven}, k+\theta }^{{\textrm{sys}}} \\ \frac{V_{\textrm{at}}^{r}\left( \varvec{d}_{k+1}\right) -V_{\textrm{at}}^{r}\left( \varvec{d}_k\right) }{\Delta t}-q_{\textrm{ven}, k+\theta }^{\textrm{sys}}+q_{\textrm{v}, \textrm{in}, k+\theta }^{r} \\ \frac{q_{\textrm{v,in},k+1}^{r} - q_{\textrm{v,in},k}^{r}}{\Delta t} + \theta \frac{ \rho \ q_{\textrm{v,in},k+1}^{r} |q_{\textrm{v,in},k+1}^{r} |- 2 \left( p_{\textrm{at},k+1}^{r} - p_{\textrm{v},k+1}^{r}\right) \left[ \left( A_{\textrm{eff,max}}^{{\textrm{tri}}} - A_{\textrm{eff,min}}^{{\textrm{tri}}} \right) \ \zeta _{k+1}^{{\textrm{tri}}} + A_{\textrm{eff,min}}^{{\textrm{tri}}}\right] ^{2} }{2 \rho \ l_{\textrm{eff}}^{{\textrm{tri}}} \left[ \left( A_{\textrm{eff,max}}^{{\textrm{tri}}} - A_{\textrm{eff,min}}^{{\textrm{tri}}} \right) \ \zeta _{k+1}^{{\textrm{tri}}} + A_{\textrm{eff,min}}^{{\textrm{tri}}}\right] } + (1-\theta ) \frac{ \rho \ q_{\textrm{v,in},k}^{r} |q_{\textrm{v,in},k}^{r} |- 2 \left( p_{\textrm{at},k}^{r} - p_{\textrm{v},k}^{r}\right) \left[ \left( A_{\textrm{eff,max}}^{{\textrm{tri}}} - A_{\textrm{eff,min}}^{{\textrm{tri}}} \right) \ \zeta _{k}^{{\textrm{tri}}} + A_{\textrm{eff,min}}^{{\textrm{tri}}}\right] ^{2} }{2 \rho \ l_{\textrm{eff}}^{{\textrm{tri}}} \left[ \left( A_{\textrm{eff,max}}^{{\textrm{tri}}} - A_{\textrm{eff,min}}^{{\textrm{tri}}} \right) \ \zeta _{k}^{{\textrm{tri}}} + A_{\textrm{eff,min}}^{{\textrm{tri}}}\right] } \\ { {\left\{ \begin{array}{ll} \frac{\zeta ^{{\textrm{tri}}}_{k+1} - \zeta ^{{\textrm{tri}}}_{k}}{\Delta t} - \theta (1-\zeta ^{{\textrm{tri}}}_{k+1}) \ K_{\textrm{v o}}^{{\textrm{tri}}} \left( p_{\textrm{at},k+1}^{r} - p_{\textrm{v},k+1}^{r}\right) - (1-\theta ) (1-\zeta ^{{\textrm{tri}}}_{k}) \ K_{\textrm{v o}}^{{\textrm{tri}}} \left( p_{\textrm{at},k}^{r} - p_{\textrm{v},k}^{r}\right) , \quad \quad & \text {if} \quad p_{\textrm{at},k+1}^{r} - p_{\textrm{v},k+1}^{r}>0, \\ \frac{\zeta ^{{\textrm{tri}}}_{k+1} - \zeta ^{{\textrm{tri}}}_{k}}{\Delta t} - \theta \zeta ^{{\textrm{tri}}}_{k+1} \ K_{\textrm{v c}}^{{\textrm{tri}}} \ \left( p_{\textrm{at},k+1}^{r} - p_{\textrm{v},k+1}^{r}\right) - (1-\theta ) \zeta ^{{\textrm{tri}}}_{k} \ K_{\textrm{v c}}^{{\textrm{tri}}} \ \left( p_{\textrm{at},k}^{r} - p_{\textrm{v},k}^{r}\right) , \quad \quad & \text {if} \quad p_{\textrm{at},k+1}^{r} - p_{\textrm{v},k+1}^{r} \le 0,\end{array}\right. }} \\ \frac{V_{\textrm{v}}^{r}\left( \varvec{d}_{k+1}\right) -V_{\textrm{v}}^{r}\left( \varvec{d}_k\right) }{\Delta t}-q_{\textrm{v,in}, k+\theta }^{r}+q_{\textrm{v,out}, k+\theta }^{r}\\ \frac{q_{\textrm{v,out}, k+1}^{r}-q_{\textrm{v,out}, k}^{r}}{\Delta t} + \theta \frac{ \rho \ q_{\textrm{v,out},k+1}^{r} |q_{\textrm{v,out},k+1}^{r} |- 2 \left( p_{\textrm{v},k+1}^{r} - p_{\textrm{ar},k+1}^{{\textrm{pul}}}\right) \left[ \left( A_{\textrm{eff,max}}^{{\textrm{pul}}} - A_{\textrm{eff,min}}^{{\textrm{pul}}} \right) \ \zeta _{k+1}^{{\textrm{pul}}} + A_{\textrm{eff,min}}^{{\textrm{pul}}}\right] ^{2} }{2 \rho \ l_{\textrm{eff}}^{{\textrm{pul}}} \left[ \left( A_{\textrm{eff,max}}^{{\textrm{pul}}} - A_{\textrm{eff,min}}^{{\textrm{pul}}} \right) \ \zeta _{k+1}^{{\textrm{pul}}} + A_{\textrm{eff,min}}^{{\textrm{pul}}}\right] } + (1-\theta ) \frac{ \rho \ q_{\textrm{v,out},k}^{r} |q_{\textrm{v,out},k}^{r} |- 2 \left( p_{\textrm{v},k}^{r} - p_{\textrm{ar},k}^{{\textrm{pul}}}\right) \left[ \left( A_{\textrm{eff,max}}^{{\textrm{pul}}} - A_{\textrm{eff,min}}^{{\textrm{pul}}} \right) \ \zeta _{k}^{{\textrm{pul}}} + A_{\textrm{eff,min}}^{{\textrm{pul}}}\right] ^{2} }{2 \rho \ l_{\textrm{eff}}^{{\textrm{pul}}} \left[ \left( A_{\textrm{eff,max}}^{{\textrm{pul}}} - A_{\textrm{eff,min}}^{{\textrm{pul}}} \right) \ \zeta _{k}^{{\textrm{pul}}} + A_{\textrm{eff,min}}^{{\textrm{pul}}}\right] } \\ { {\left\{ \begin{array}{ll} \frac{\zeta ^{{\textrm{pul}}}_{k+1} - \zeta ^{{\textrm{pul}}}_{k}}{\Delta t} - \theta (1-\zeta 
^{{\textrm{pul}}}_{k+1}) \ K_{\textrm{v o}}^{{\textrm{pul}}} \left( p_{\textrm{v},k+1}^{r} - p_{\textrm{ar},k+1}^{{\textrm{pul}}}\right) - (1-\theta ) (1-\zeta ^{{\textrm{pul}}}_{k}) \ K_{\textrm{v o}}^{{\textrm{pul}}} \left( p_{\textrm{v},k}^{r} - p_{\textrm{ar},k}^{{\textrm{pul}}}\right) , \quad \quad & \text {if} \quad p_{\textrm{v},k+1}^{r} - p_{\textrm{ar},k+1}^{{\textrm{pul}}}>0, \\ \frac{\zeta ^{{\textrm{pul}}}_{k+1} - \zeta ^{{\textrm{pul}}}_{k}}{\Delta t} - \theta \zeta ^{{\textrm{pul}}}_{k+1} \ K_{\textrm{v c}}^{{\textrm{pul}}} \ \left( p_{\textrm{v},k+1}^{r} - p_{\textrm{ar},k+1}^{{\textrm{pul}}}\right) - (1-\theta ) \zeta ^{{\textrm{pul}}}_{k} \ K_{\textrm{v c}}^{{\textrm{pul}}} \ \left( p_{\textrm{v},k}^{r} - p_{\textrm{ar},k}^{{\textrm{pul}}}\right) , \quad \quad & \text {if} \quad p_{\textrm{v},k+1}^{r} - p_{\textrm{ar},k+1}^{{\textrm{pul}}} \le 0,\end{array}\right. }} \\ C_{\textrm{ar}}^{{\textrm{pul}}}\left( \frac{p_{\textrm{ar}, k+1}^{{\textrm{pul}}}-p_{\textrm{ar}, k}^{{\textrm{pul}}}}{\Delta t}-Z_{\textrm{ar}}^{{\textrm{pul}}} \frac{q_{\textrm{v,out}, k+1}^{r}-q_{\textrm{v,out}, k}^{r}}{\Delta t}\right) -q_{\textrm{v,out}, k+\theta }^{r}+q_{\textrm{ar}, k+\theta }^{{\textrm{pul}}} \\ \frac{1}{R_{\textrm{ar}}^{{\textrm{pul}}}} \left( p_{\textrm{ven}, k+\theta }^{{\textrm{pul}}} - p_{\textrm{ar}, k+\theta }^{{\textrm{pul}}} + Z_{\textrm{ar}}^{{\textrm{pul}}} q_{\textrm{v,out}, k+\theta }^{r}\right) + q_{\textrm{ar}, k+\theta }^{{\textrm{pul}}} \\ C_{\textrm{ven}}^{{\textrm{pul}}}\frac{p_{\textrm{ven}, k+1}^{{\textrm{pul}}}-p_{\textrm{ven}, k}^{{\textrm{pul}}}}{\Delta t}-q_{\textrm{ar}, k+\theta }^{{\textrm{pul}}} + q_{\textrm{ven}, k+\theta }^{{\textrm{pul}}}\\ \frac{1}{R_{\textrm{ven}}^{{\textrm{pul}}}} \left( p_{\textrm{at}, k+\theta }^{\ell } - p_{\textrm{ven}, k+\theta }^{{\textrm{pul}}} \right) + q_{\textrm{ven}, k+\theta }^{{\textrm{pul}}} \\ \end{bmatrix} \end{aligned} \end{aligned}$$

where $$(\bullet )_{k+\theta } = \theta \ (\bullet )_{k+1}+ (1-\theta )(\bullet )_{k}$$.

## Path-dependent adjoint sensitivities for the 3D–0D coupled model

In order to solve the inverse problem stated in Equation (49), the computation of the gradient information of the objective function $f_{\tau }(\varvec{\phi })$ depicted in (51) with respect to the vector of active stress parameters $\varvec{\phi }$ is required. The objective function $f_{\tau }$ depends implicitly on the parameters $\varvec{\phi }$ to be identified, as its evaluation requires the values of the 3D primary variables $\varvec{d}$, which are solution of the nonlinear system of coupled equations (A) and (29). In contrast to the forward problem, the time discretization equations

B1 $$\begin{aligned}&\begin{aligned} \varvec{R}^{v}_{k} =&\varvec{v}_{k+1} - \frac{\gamma }{\beta \Delta t}\left( \varvec{d}_{k+1}-\varvec{d}_k\right) + \frac{\gamma -\beta }{\beta } \ \varvec{v}_k \\ &+ \frac{\gamma -2 \beta }{2 \beta } \Delta t \ \varvec{a}_k=\varvec{0}, \end{aligned} \end{aligned}$$

B2 $$\begin{aligned}&\begin{aligned} \varvec{R}^{a}_{k} =&\varvec{a}_{k+1} - \frac{1}{\beta \Delta t^2}\left( \varvec{d}_{k+1} + \varvec{d}_k\right) -\frac{1}{\beta \Delta t} \ \varvec{v}_k \\ &+ \frac{1-2 \beta }{2 \beta } \ \varvec{a}_k=\varvec{0}, \end{aligned} \end{aligned}$$

are considered explicitly in the path-dependent adjoint problem to avoid tedious chain rule derivatives as proposed by Alberdi et al (2018). Therefore, the global residual of the nonlinear coupled governing equations is expressed as

B3 $$\begin{aligned} \begin{aligned}&\varvec{R}_{k}(\varvec{\phi },\varvec{x}_{k}, \varvec{x}_{k-1}) = \\&\begin{bmatrix} \varvec{R}^{\mathrm{{3D}}}_{k}(\varvec{\phi },\varvec{d}_{k},\varvec{v}_{k},\varvec{a}_{k},\varvec{q}_{k}) \\ \varvec{R}^{v}_{k}(\varvec{d}_{k},\varvec{v}_{k},\varvec{d}_{k-1},\varvec{v}_{k-1},\varvec{a}_{k-1}) \\ \varvec{R}^{a}_{k}(\varvec{d}_{k},\varvec{a}_{k},\varvec{d}_{k-1},\varvec{v}_{k-1},\varvec{a}_{k-1}) \\ \varvec{R}^{\mathrm{{0D}}}_{k}(\varvec{d}_{k}, \varvec{d}_{k-1},\varvec{q}_{k}, \varvec{q}_{k-1}) \end{bmatrix} = \varvec{0}, \\&k =1,2, \ \dots \, n, \end{aligned} \end{aligned}$$

with the global vector of primary variables $\varvec{x}_{k} = [\varvec{d}_{k} \ \varvec{v}_{k} \ \varvec{a}_{k} \ \varvec{q}_{k}]^{T}$. The residual vector of the equations of motion of the 3D submodel $\varvec{R}^{\mathrm{{3D}}}_{k}$ is considered here at the endpoint of time step *k* as

B4 $$\begin{aligned} \begin{aligned} \varvec{R}^{\mathrm{{3D}}}_{k} =&\varvec{M}\ \varvec{a}_{k} + \varvec{C}\ \varvec{v}_{k} + \varvec{F}_{\textrm{int},k}(\varvec{\phi },\varvec{d}_{k}) \\ &- \varvec{F}_{\textrm{ext},k}(\varvec{d}_{k},\varvec{v}_{k},\varvec{q}_{k}) = \varvec{0}, \end{aligned} \end{aligned}$$

where the internal force vector $\varvec{F}_{\textrm{int},k}$ is dependent on the active stress parameters $\varvec{\phi }$, since they govern the active stress function $\tau _{a}$ in the stress tensor (14). The blocks $\varvec{R}^{v}_{k}$ and $\varvec{R}^{a}_{k}$ are associated with the residuals of the time discretization approximations (Chung and Hulbert 1993). The last block $\varvec{R}^{\mathrm{{0D}}}_{k}$ defines the residual vector of the 0D submodel (A).

Although the objective function $f_{\tau }$ depicted in (51) depends only on a subset of 3D variables $\varvec{d}$ at a selected number of time steps, a dependency on the entire history of the global vector of variables $\varvec{x}$ is assumed here for notation conciseness

B5 $$\begin{aligned} f_{\tau }(\varvec{\phi }) = \mathcal {F} (\varvec{x}_{1} (\varvec{\phi }), \, \dots \,, \varvec{x}_{n} (\varvec{\phi }) ), \end{aligned}$$

where the variables $\varvec{x}$ depend on the parameters $\varvec{\phi }$ based on the governing equations (B3). Note that even if the objective function is only dependent on the solution at a single time step $\bar{k}$, the sensitivity analysis of a path-dependent problem, such as the dynamic problem considered in this work, must take into account the entire previous solution history of all time steps $k<\bar{k}$ (Alberdi et al 2018).

In order to evaluate the gradient $\frac{\textrm{d} \, f_{\tau }}{\textrm{d} \varvec{\phi }}$ efficiently by means of the adjoint equations as proposed by Alberdi et al (2018); Michaleris et al (1994), we construct a Lagrangian function

B6 $$\begin{aligned} \begin{aligned} \hat{f}_{\tau }(\varvec{\phi })= f_{\tau }(\varvec{\phi }) + \sum _{k=1}^{n} \varvec{\lambda }_{k}^{T} \ \varvec{R}_{k}, \end{aligned} \end{aligned}$$

where $\varvec{\lambda }_{k}$ are the Lagrange multipliers associated with the residual of the governing equations $\varvec{R}_{k}~=~\varvec{0}$. The expansion of $\frac{\textrm{d} \, f_{\tau }}{\textrm{d} \varvec{\phi }}$ using the chain rule yields

B7 $$\begin{aligned} \begin{aligned}&\frac{\textrm{d} \, f_{\tau }}{\textrm{d} \varvec{\phi }} = \frac{\textrm{d} \, \hat{f}_{\tau }}{\textrm{d} \varvec{\phi }} = \sum _{k=1}^{n} \frac{\partial \mathcal {F}}{\partial \varvec{x}_{k}} \frac{\textrm{d} \varvec{x}_{k}}{\textrm{d} \varvec{\phi }} \\ &+ \sum _{k=1}^{n} \varvec{\lambda }_{k}^{T} \left( \frac{\partial \varvec{R}_{k}}{\partial \varvec{\phi }} + \frac{\partial \varvec{R}_{k}}{\partial \varvec{x}_{k}} \frac{\textrm{d} \varvec{x}_{k}}{\textrm{d} \varvec{\phi }} + \frac{\partial \varvec{R}_{k}}{\partial \varvec{x}_{k-1}} \frac{\textrm{d} \varvec{x}_{k-1}}{\textrm{d} \varvec{\phi }}\right) . \end{aligned} \end{aligned}$$

where the term $\frac{\partial \varvec{\lambda }_{k}}{\partial \varvec{\phi }}$ is eliminated, since it is multiplied with $\varvec{R}_{k}~=~\varvec{0}$. The recombination of Equation (B7) by gathering all the terms that contain the same implicit derivative $\frac{\textrm{d} \varvec{x}_{k}}{\textrm{d} \varvec{\phi }}$ reads

B8 $$\begin{aligned} \begin{aligned}&\frac{\textrm{d} \, \hat{f}_{\tau }}{\textrm{d} \varvec{\phi }} = \left[ \sum _{k=1}^{n} \varvec{\lambda }_{k}^{T} \frac{\partial \varvec{R}_{k}}{\partial \varvec{\phi }} \right] + \left( \frac{\partial \mathcal {F}}{\partial \varvec{x}_{n}} + \varvec{\lambda }_{n}^{T} \frac{\partial \varvec{R}_{n}}{\partial \varvec{x}_{n}} \right) \frac{\textrm{d} \varvec{x}_{n}}{\textrm{d} \varvec{\phi }} \\ &+ \sum _{k=1}^{n-1} \left( \frac{\partial \mathcal {F}}{\partial \varvec{x}_{k}} + \varvec{\lambda }_{k+1}^{T} \frac{\partial \varvec{R}_{k+1}}{\partial \varvec{x}_{k}} + \varvec{\lambda }_{k}^{T} \frac{\partial \varvec{R}_{k}}{\partial \varvec{x}_{k}} \right) \frac{\textrm{d} \varvec{x}_{k}}{\textrm{d} \varvec{\phi }}. \end{aligned} \end{aligned}$$

The implicit derivatives $\frac{\textrm{d} \varvec{x}_{k}}{\textrm{d} \varvec{\phi }}$ are then eliminated by defining the Lagrange multipliers $\varvec{\lambda }_{k}$ as solutions of the following adjoint equations

B9 $$\begin{aligned} \begin{aligned} n \text {th step}:&\quad \frac{\partial \mathcal {F}}{\partial \varvec{x}_{n}} + \varvec{\lambda }_{n}^{T} \frac{\partial \varvec{R}_{n}}{\partial \varvec{x}_{n}} = \varvec{0}, \\ k \text {th step}:&\quad \frac{\partial \mathcal {F}}{\partial \varvec{x}_{k}} + \varvec{\lambda }_{k+1}^{T} \frac{\partial \varvec{R}_{k+1}}{\partial \varvec{x}_{k}} + \varvec{\lambda }_{k}^{T} \frac{\partial \varvec{R}_{k}}{\partial \varvec{x}_{k}} = \varvec{0}, \\ k =&n-1, \ \dots \, 2,1, \end{aligned} \end{aligned}$$

which is solved in a backward order, in contrast to the forward problem, meaning that the solution of the systems of equations in (B9) starts with the last step *n* and ends with the first step. After solving the adjoint equations, the gradient is evaluated as

B10 $$\begin{aligned} \frac{\textrm{d} \, f_{\tau }}{\textrm{d} \varvec{\phi }} = \frac{\textrm{d} \, \hat{f}_{\tau }}{\textrm{d} \varvec{\phi }} = \sum _{k=1}^{n} \varvec{\lambda }_{k}^{T} \frac{\partial \varvec{R}_{k}}{\partial \varvec{\phi }}. \end{aligned}$$

## Spatial convergence analysis

A spatial convergence study is performed to assess the sensitivity of the simulation results to the spatial discretization of the 3D domain. All meshes are generated using 4-node tetrahedral elements with average element sizes of 1.5 mm, 2 mm, 2.5 mm, 3 mm, and 4 mm. The calibrated model, with the patient-specific parameters listed in Tables 7 and 8, is used for this convergence analysis to ensure consistency with the main simulation results. The output quantities of interest are the ventricular volumes and pressures, presented as pressure-volume (PV) loops for both the left and right ventricles in Fig. 19. These PV loops comprehensively characterize the global mechanical response of the heart chambers. Quantitative comparisons of the PV loops across the different mesh resolutions show that the differences in end-diastolic and end-systolic volumes and pressures decrease as the mesh is refined. For meshes finer than 2.5 mm, the variations in these key quantities remain below 2 ml amd 2 mmHg. Notably, the left ventricular quantities exhibit slower convergence compared to the right ventricle, but overall convergence is satisfactory for the accuracy required in this application. For each mesh resolution, the number of elements and variables, as well as maximum and minimum volume and pressure values, are listed in Table 10. Based on these results, an average element size of 2.5 mm is selected for all performed simulations, as it ensures numerical accuracy while maintaining computational efficiency.

**Table 10:** Maximum and minimum volume and pressure values of left and right ventricles for different average element sizes, sorted ascendingly with respect to the number of variables

Elem. Size	# Elements	# Variables	$V^{\ell }_{\textrm{max}}$[ml]	$V^{\ell }_{\textrm{min}}$[ml]	$V^{r}_{\textrm{max}}$[ml]	$V^{r}_{\textrm{min}}$[ml]	$p^{\ell }_{\textrm{v,max}}$[mmHg]	$p^{\ell }_{\textrm{v,min}}$[mmHg]	$p^{r}_{\textrm{v,max}}$[mmHg]	$p^{r}_{\textrm{v,min}}$[mmHg]
1.5 mm	447,631	287,160	102.1	60.4	195.9	115.9	96.2	2.1	33.4	3.1
2.0 mm	189,322	130,713	101.2	60.7	195.6	116.0	95.1	2.1	33.4	3.2
2.5 mm	97,737	71,838	100.2	60.5	195.1	116.6	94.6	2.0	32.5	3.3
3.0 mm	59,806	46,026	99.2	60.5	194.5	116.6	93.6	1.8	32.6	3.4
4.0 mm	28,491	23,562	98.0	60.6	193.8	118.5	92.5	1.6	31.9	3.5

## References

[CR1] Alber M, Buganza Tepole A, Cannon WR et al (2019) Integrating machine learning and multiscale modeling-perspectives, challenges, and opportunities in the biological, biomedical, and behavioral sciences. npj Digit Med 2(1):115. 10.1038/s41746-019-0193-y 31799423 10.1038/s41746-019-0193-y PMC6877584

[CR2] Alberdi R, Zhang G, Li L et al (2018) A unified framework for nonlinear path-dependent sensitivity analysis in topology optimization. Int J Numer Meth Eng 115(1):1–56. 10.1002/nme.5794

[CR3] Aletras AH, Ding S, Balaban RS et al (1999) DENSE: displacement Encoding with Stimulated Echoes in Cardiac Functional MRI. J Magn Reson 137(1):247–252. 10.1006/jmre.1998.1676 10053155 10.1006/jmre.1998.1676 PMC2887318

[CR4] Asner L, Hadjicharalambous M, Chabiniok R et al (2016) Estimation of passive and active properties in the human heart using 3D tagged MRI. Biomech Model Mechanobiol 15(5):1121–1139. 10.1007/s10237-015-0748-z 26611908 10.1007/s10237-015-0748-z PMC5021775

[CR5] Balaban G, Finsberg H, Funke S et al (2018) In vivo estimation of elastic heterogeneity in an infarcted human heart. Biomech Model Mechanobiol 17(5):1317–1329. 10.1007/s10237-018-1028-5 29774440 10.1007/s10237-018-1028-5 PMC6154126

[CR6] Barclay GJ, Griffiths DF, Higham DJ (2000) Theta method dynamics. LMS J Comput Math 3:27–43. 10.1112/S146115700000019X

[CR7] Bertoglio C, Barber D, Gaddum N et al (2014) Identification of artery wall stiffness: in vitro validation and in vivo results of a data assimilation procedure applied to a 3D fluid-structure interaction model. J Biomech 47(5):1027–1034. 10.1016/j.jbiomech.2013.12.029 24529756 10.1016/j.jbiomech.2013.12.029

[CR8] Bestel J, Clément F, Sorine M (2001) A biomechanical model of muscle contraction. In: Goos G, Hartmanis J, Van Leeuwen J, et al (eds.) Medical Image Computing and Computer-Assisted Intervention ??? MICCAI 2001, vol 2208. Springer Berlin Heidelberg, Berlin, Heidelberg, p 1159–1161, 10.1007/3-540-45468-3_143,

[CR9] Bracamonte JH, Saunders SK, Wilson JS et al (2022) Patient-specific inverse modeling of in vivo cardiovascular mechanics with medical image-derived kinematics as input data: concepts, methods, and applications. Appl Sci 12(8):3954. 10.3390/app12083954 36911244 10.3390/app12083954 PMC10004130

[CR10] Broyden CG (1970) The convergence of a class of double-rank minimization algorithms 1. General considerations. IMA J Appl Math 6(1):76–90. 10.1093/imamat/6.1.76

[CR11] Caruel M, Chabiniok R, Moireau P et al (2014) Dimensional reductions of a cardiac model for effective validation and calibration. Biomech Model Mechanobiol 13(4):897–914. 10.1007/s10237-013-0544-6 24317551 10.1007/s10237-013-0544-6

[CR12] Chapelle D, Le Gall A (2023) A biomechanics-based parametrized cardiac end-diastolic pressure-volume relationship for accurate patient-specific calibration and estimation. Sci Rep 13(1):11232. 10.1038/s41598-023-38196-5 37433813 10.1038/s41598-023-38196-5 PMC10336140

[CR13] Chung J, Hulbert GM (1993) A time integration algorithm for structural dynamics with improved numerical dissipation: the generalized-\$alpha\$ method. J Appl Mech 60(2):371–375. 10.1115/1.2900803

[CR14] Coorey G, Figtree GA, Fletcher DF et al (2022) The health digital twin to tackle cardiovascular disease-a review of an emerging interdisciplinary field. npj Digit Med 5(1):126. 10.1038/s41746-022-00640-7 36028526 10.1038/s41746-022-00640-7 PMC9418270

[CR15] Corral-Acero J, Margara F, Marciniak M et al (2020) The???-Digital Twin’ to enable the vision of precision cardiology. Eur Heart J 41(48):4556–4564. 10.1093/eurheartj/ehaa159 32128588 10.1093/eurheartj/ehaa159 PMC7774470

[CR16] D’Alto M, Dimopoulos K, Budts W et al (2016) Multimodality imaging in congenital heart disease-related pulmonary arterial hypertension. Heart 102(12):910–918. 10.1136/heartjnl-2015-308903 27013702 10.1136/heartjnl-2015-308903

[CR17] Datz JC, Steinbrecher I, Meier C et al (2024) Patient-specific coronary angioplasty simulations–a mixed-dimensional finite element modeling approach. Comput Biol Med 189:109914 10.1016/j.compbiomed.2025.109914 PMC13238462 40068490

[CR18] De Vecchi A, Gomez A, Pushparajah K et al (2016) A novel methodology for personalized simulations of ventricular hemodynamics from noninvasive imaging data. Comput Med Imaging Graph 51:20–31. 10.1016/j.compmedimag.2016.03.004 27108088 10.1016/j.compmedimag.2016.03.004 PMC4907311

[CR19] Del Rio-Pertuz G, Nugent K, Argueta-Sosa E (2023) Right heart catheterization in clinical practice: a review of basic physiology and important issues relevant to interpretation. Am J Cardiovascular Dis 13(3):122–137 PMC10352814 37469534

[CR20] Doste R, Soto-Iglesias D, Bernardino G et al (2019) A rule-based method to model myocardial fiber orientation in cardiac biventricular geometries with outflow tracts. Int J Num Methods Biomed Eng 35(4):e3185. 10.1002/cnm.3185 10.1002/cnm.3185 30721579

[CR21] Fedele M, Piersanti R, Regazzoni F et al (2023) A comprehensive and biophysically detailed computational model of the whole human heart electromechanics. Comput Methods Appl Mech Eng 410:115983. 10.1016/j.cma.2023.115983

[CR22] Finsberg H, Balaban G, Ross S et al (2018) Estimating cardiac contraction through high resolution data assimilation of a personalized mechanical model. J Comput Sci 24:85–90. 10.1016/j.jocs.2017.07.013

[CR23] Flachskampf FA, Rodriguez L, Chen C et al (1993) Analysis of mitral inertance: a factor critical for early transmitral filling. J Am Soc Echocardiogr 6(4):422–432. 10.1016/S0894-7317(14)80241-1 8217209 10.1016/s0894-7317(14)80241-1

[CR24] Fletcher R (1970) A new approach to variable metric algorithms. Comput J 13(3):317–322. 10.1093/comjnl/13.3.317

[CR25] Fratz S, Schuhbaeck A, Buchner C et al (2009) Comparison of accuracy of axial slices versus short-axis slices for measuring ventricular volumes by cardiac magnetic resonance in patients with corrected tetralogy of fallot. Am J Cardiol 103(12):1764–1769. 10.1016/j.amjcard.2009.02.030 19539090 10.1016/j.amjcard.2009.02.030

[CR26] Fumagalli I, Pagani S, Vergara C, et al (2024) The role of computational methods in cardiovascular medicine: a narrative review. Trans Pediatrics. 13(1):146–163. 10.21037/tp-23-184 10.21037/tp-23-184 PMC10839285 38323181

[CR27] Ka G, Taylor A, Owens L et al (1967) Pulmonary capillary pressure and filtration coefficient in the isolated perfused lung. Am J Physiol-Legacy Content 213(4):910–914. 10.1152/ajplegacy.1967.213.4.910 10.1152/ajplegacy.1967.213.4.910 6051189

[CR28] Garcia D, Kadem L (2006) What do you mean by aortic valve area: geometric orifice area, effective orifice area, or gorlin area? J Heart Valve Dis 15(5):601–608 17044363

[CR29] Garcia D, Pibarot P, Durand LG (2005) Analytical modeling of the instantaneous pressure gradient across the aortic valve. J Biomech 38(6):1303–1311. 10.1016/j.jbiomech.2004.06.018 15863115 10.1016/j.jbiomech.2004.06.018

[CR30] Gee MW, Förster C, Wall WA (2010) A computational strategy for prestressing patient-specific biomechanical problems under finite deformation. Int J Num Methods Biomed Eng 26(1):52–72. 10.1002/cnm.1236

[CR31] Gelman S, Warner D, Warner M (2008) Venous function and central venous pressure. Anesthesiology 108(4):735–748. 10.1097/ALN.0b013e3181672607 18362606 10.1097/ALN.0b013e3181672607

[CR32] Genet M, Lee LC, Nguyen R et al (2014) Distribution of normal human left ventricular myofiber stress at end diastole and end systole: a target for in silico design of heart failure treatments. J Appl Physiol 117(2):142–152. 10.1152/japplphysiol.00255.2014 24876359 10.1152/japplphysiol.00255.2014 PMC4101610

[CR33] Genet M, Lee LC, Baillargeon B et al (2016) Modeling pathologies of diastolic and systolic heart failure. Ann Biomed Eng 44(1):112–127. 10.1007/s10439-015-1351-2 26043672 10.1007/s10439-015-1351-2 PMC4670609

[CR34] Georgiev S, Ewert P, Eicken A et al (2020) Munich comparative study: prospective long-term outcome of the transcatheter melody valve versus surgical pulmonary bioprosthesis with up to 12 years of follow-up. Circ Cardiovasc Interv 13(7):e008963 32600110 10.1161/CIRCINTERVENTIONS.119.008963

[CR35] Gerach T, Schuler S, Fröhlich J et al (2021) Electro-mechanical whole-heart digital twins: a fully coupled multi-physics approach. Mathematics 9(11):1247. 10.3390/math9111247

[CR36] Ghadimi S, Abdi M, Epstein FH (2023) Improved computation of Lagrangian tissue displacement and strain for cine DENSE MRI using a regularized spatiotemporal least squares method. Front Cardiovascular Med 10:1095159. 10.3389/fcvm.2023.1095159 10.3389/fcvm.2023.1095159 PMC10061004 37008315

[CR37] Goldfarb D (1970) A family of variable-metric methods derived by variational means. Math Comput 24(109):23–26. 10.1090/S0025-5718-1970-0258249-6

[CR38] Hadjicharalambous M, Stoeck CT, Weisskopf M et al (2021) Investigating the reference domain influence in personalised models of cardiac mechanics: effect of unloaded geometry on cardiac biomechanics. Biomech Model Mechanobiol 20(4):1579–1597. 10.1007/s10237-021-01464-2 34047891 10.1007/s10237-021-01464-2

[CR39] Hirschvogel M, Bassilious M, Jagschies L et al (2017) A monolithic 3D–0D coupled closed-loop model of the heart and the vascular system: experiment-based parameter estimation for patient-specific cardiac mechanics. Int J Num Methods Biomed Eng 33(8):e2842. 10.1002/cnm.2842 10.1002/cnm.2842 27743468

[CR40] Hirschvogel M, Jagschies L, Maier A et al (2019) An in silico twin for epicardial augmentation of the failing heart. Int J Num Methods Biomed Eng 35(10):e3233. 10.1002/cnm.3233 10.1002/cnm.3233 31267697

[CR41] Holzapfel GA, Ogden RW (2009) Constitutive modelling of passive myocardium: a structurally based framework for material characterization. Philosophical Trans Royal Soc Math Phys Eng Sci 367(1902):3445–3475. 10.1098/rsta.2009.0091 10.1098/rsta.2009.0091 19657007

[CR42] Hose DR, Lawford PV, Huberts W et al (2019) Cardiovascular models for personalised medicine: Where now and where next? Med Eng Phys 72:38–48. 10.1016/j.medengphy.2019.08.007 31554575 10.1016/j.medengphy.2019.08.007

[CR43] Imperiale A, Chapelle D, Moireau P (2021) Sequential data assimilation for mechanical systems with complex image data: application to tagged-MRI in cardiac mechanics. Adv Model Simul Eng Sci 8(1):2. 10.1186/s40323-020-00179-w

[CR44] Ismail M, Wall WA, Gee MW (2013) Adjoint-based inverse analysis of windkessel parameters for patient-specific vascular models. J Comput Phys 244:113–130. 10.1016/j.jcp.2012.10.028

[CR45] Itu L, Sharma P, Passerini T et al (2015) A parameter estimation framework for patient-specific hemodynamic computations. J Comput Phys 281:316–333. 10.1016/j.jcp.2014.10.034

[CR46] Kehl S, Gee MW (2017) Calibration of parameters for cardiovascular models with application to arterial growth. Int J Num Methods Biomed Eng 33(5):e2822. 10.1002/cnm.2822 10.1002/cnm.2822 27501849

[CR47] Land S, Gurev V, Arens S et al (2015) Verification of cardiac mechanics software: benchmark problems and solutions for testing active and passive material behaviour. Proceed Royal Soc Math Phys Eng Sci 471(2184):20150641. 10.1098/rspa.2015.0641 10.1098/rspa.2015.0641 PMC4707707 26807042

[CR48] Lazarus A, Dalton D, Husmeier D et al (2022) Sensitivity analysis and inverse uncertainty quantification for the left ventricular passive mechanics. Biomech Model Mechanobiol 21(3):953–982. 10.1007/s10237-022-01571-8 35377030 10.1007/s10237-022-01571-8 PMC9132878

[CR49] Marchesseau S, Delingette H, Sermesant M et al (2013) Personalization of a cardiac electromechanical model using reduced order unscented Kalman filtering from regional volumes. Med Image Anal 17(7):816–829. 10.1016/j.media.2013.04.012 23707227 10.1016/j.media.2013.04.012

[CR50] Marsden AL (2014) Optimization in cardiovascular modeling. Annu Rev Fluid Mech 46(1):519–546. 10.1146/annurev-fluid-010313-141341

[CR51] Marsden AL, Esmaily-Moghadam M (2015) Multiscale modeling of cardiovascular flows for clinical decision support. Appl Mech Rev 67(3):030804. 10.1115/1.4029909

[CR52] MATLAB (2022) MATLAB version: 9.13.0 (R2022b). https://www.mathworks.com

[CR53] Meierhofer C, Lyko C, Schneider EP et al (2015) Baseline correction does not improve flow quantification in phase-contrast velocity measurement for routine clinical practice. Clin Imaging 39(3):427–431. 10.1016/j.clinimag.2014.12.010 25661574 10.1016/j.clinimag.2014.12.010

[CR54] Miao H, Xia X, Perelson AS et al (2011) On identifiability of nonlinear ode models and applications in viral dynamics. SIAM Rev 53(1):3–39. 10.1137/090757009 10.1137/090757009 PMC3140286 21785515

[CR55] Michaleris P, Tortorelli DA, Vidal CA (1994) Tangent operators and design sensitivity formulations for transient non-linear coupled problems with applications to elastoplasticity. Int J Numer Meth Eng 37(14):2471–2499. 10.1002/nme.1620371408

[CR56] Molléro R, Pennec X, Delingette H et al (2018) Multifidelity-CMA: a multifidelity approach for efficient personalisation of 3D cardiac electromechanical models. Biomech Model Mechanobiol 17(1):285–300. 10.1007/s10237-017-0960-0 28894984 10.1007/s10237-017-0960-0

[CR57] Mondillo S, Galderisi M, Mele D et al (2011) Speckle-tracking echocardiography: a new technique for assessing myocardial function. J Ultrasound Med 30(1):71–83. 10.7863/jum.2011.30.1.71 21193707 10.7863/jum.2011.30.1.71

[CR58] Mynard JP, Davidson MR, Penny DJ et al (2012) A simple, versatile valve model for use in lumped parameter and one-dimensional cardiovascular models. Int J Num Methods Biomed Eng 28(6–7):626–641. 10.1002/cnm.1466 10.1002/cnm.1466 25364842

[CR59] Nair PJ, Pfaller MR, Dual SA, et al (2023) Non-invasive estimation of pressure drop across aortic coarctations: validation of 0D and 3D computational models with in vivo measurements. medRxiv: The Preprint Server for Health Sciences p 2023.09.05.23295066. 10.1101/2023.09.05.23295066 10.1007/s10439-024-03457-5 PMC13271072 38341399

[CR60] Niederer SA, Lumens J, Trayanova NA (2019) Computational models in cardiology. Nat Rev Cardiol 16(2):100–111. 10.1038/s41569-018-0104-y 30361497 10.1038/s41569-018-0104-y PMC6556062

[CR61] Nocedal J, Wright SJ (1999) Numerical optimization. Springer series in operations research, Springer, New York

[CR62] Nolte D, Bertoglio C (2022) Inverse problems in blood flow modeling: a review. Int J Num Methods Biomed Eng 38(8):e3613. 10.1002/cnm.3613 10.1002/cnm.3613 PMC9541505 35526113

[CR63] Nordsletten D, Niederer S, Nash M et al (2011) Coupling multi-physics models to cardiac mechanics. Prog Biophys Mol Biol 104(1–3):77–88. 10.1016/j.pbiomolbio.2009.11.001 19917304 10.1016/j.pbiomolbio.2009.11.001

[CR64] Oberai AA, Gokhale NH, Feijo GR (2003) Solution of inverse problems in elasticity imaging using the adjoint method. Inverse Prob 19(2):297–313. 10.1088/0266-5611/19/2/304. (**publisher: IOP Publishing**)

[CR65] Pant S, Corsini C, Baker C et al (2017) Inverse problems in reduced order models of cardiovascular haemodynamics: aspects of data assimilation and heart rate variability. J R Soc Interface 14(126):20160513. 10.1098/rsif.2016.0513 28077762 10.1098/rsif.2016.0513 PMC5310725

[CR66] Peirlinck M, Costabal FS, Yao J et al (2021) Precision medicine in human heart modeling: Perspectives, challenges, and opportunities. Biomech Model Mechanobiol 20(3):803–831. 10.1007/s10237-021-01421-z 33580313 10.1007/s10237-021-01421-z PMC8154814

[CR67] Perego M, Veneziani A, Vergara C (2011) A variational approach for estimating the compliance of the cardiovascular tissue: an inverse fluid-structure interaction problem. SIAM J Sci Comput 33(3):1181–1211. 10.1137/100808277. (**publisher: Society for Industrial & Applied Mathematics (SIAM)**)

[CR68] Pfaller MR, Hörmann JM, Weigl M et al (2019) The importance of the pericardium for cardiac biomechanics: from physiology to computational modeling. Biomech Model Mechanobiol 18(2):503–529. 10.1007/s10237-018-1098-4 30535650 10.1007/s10237-018-1098-4

[CR69] Piersanti R, Africa PC, Fedele M et al (2021) Modeling cardiac muscle fibers in ventricular and atrial electrophysiology simulations. Comput Methods Appl Mech Eng 373:113468

[CR70] Piersanti R, Regazzoni F, Salvador M et al (2022) 3D???0D closed-loop model for the simulation of cardiac biventricular electromechanics. Comput Methods Appl Mech Eng 391:114607. 10.1016/j.cma.2022.114607

[CR71] Quarteroni A, Rozza G (2003) Optimal control and shape optimization of aorto-coronaric bypass anastomoses. Math Models Methods Appl Sci 13(12):1801–1823. 10.1142/s0218202503003124. (**publisher: World Scientific Pub Co Pte Ltd**)

[CR72] Regazzoni F, Salvador M, Africa P et al (2022) A cardiac electromechanical model coupled with a lumped-parameter model for closed-loop blood circulation. J Comput Phys 457:111083. 10.1016/j.jcp.2022.111083

[CR73] Revie JA, Stevenson DJ, Chase JG et al (2013) Validation of subject-specific cardiovascular system models from porcine measurements. Comput Methods Programs Biomed 109(2):197–210. 10.1016/j.cmpb.2011.10.013 22126892 10.1016/j.cmpb.2011.10.013

[CR74] Rumindo GK, Ohayon J, Croisille P et al (2020) In vivo estimation of normal left ventricular stiffness and contractility based on routine cine MR acquisition. Med Eng Phys 85:16–26. 10.1016/j.medengphy.2020.09.003 33081960 10.1016/j.medengphy.2020.09.003

[CR75] Rutz T, Ghandour F, Meierhofer C et al (2017) Evolution of right ventricular size over time after tetralogy of Fallot repair: a longitudinal cardiac magnetic resonance study. Eur Heart J - Cardiovascular Imaging 18(3):364–370. 10.1093/ehjci/jew273 10.1093/ehjci/jew273 28363200

[CR76] Saltelli A (2008) Global sensitivity analysis: the primer. John Wiley Chichester England Hoboken NJ. 10.1002/9780470725184

[CR77] Salvador M, Regazzoni F, Dede’ L et al (2023) Fast and robust parameter estimation with uncertainty quantification for the cardiac function. Comput Methods Programs Biomed 231:107402. 10.1016/j.cmpb.2023.107402 36773593 10.1016/j.cmpb.2023.107402

[CR78] Schiavazzi DE, Baretta A, Pennati G et al (2017) Patient-specific parameter estimation in single-ventricle lumped circulation models under uncertainty. Int J Num Methods Biomed Eng 33(3):e02799. 10.1002/cnm.2799 10.1002/cnm.2799 PMC5499984 27155892

[CR79] Sermesant M, Delingette H, Ayache N (2006) An electromechanical model of the heart for image analysis and simulation. IEEE Trans Med Imaging 25(5):612–625. 10.1109/TMI.2006.872746 16689265 10.1109/TMI.2006.872746

[CR80] Shanno DF (1970) Conditioning of quasi-Newton methods for function minimization. Math Comput 24(111):647–656. 10.1090/S0025-5718-1970-0274029-X

[CR81] Smith N, De Vecchi A, McCormick M et al (2011) euHeart: personalized and integrated cardiac care using patient-specific cardiovascular modelling. Interface Focus 1(3):349–364. 10.1098/rsfs.2010.0048 22670205 10.1098/rsfs.2010.0048 PMC3262448

[CR82] Spilker RL, Taylor CA (2010) Tuning multidomain hemodynamic simulations to match physiological measurements. Ann Biomed Eng 38(8):2635–2648. 10.1007/s10439-010-0011-9 20352338 10.1007/s10439-010-0011-9

[CR83] Stergiopulos N, Westerhof BE, Westerhof N (1999) Total arterial inertance as the fourth element of the windkessel model. Am J Physiol-Heart Circulatory Physiol 276(1):H81–H88. 10.1152/ajpheart.1999.276.1.H81 10.1152/ajpheart.1999.276.1.H81 9887020

[CR84] Strocchi M, Gsell MA, Augustin CM et al (2020) Simulating ventricular systolic motion in a four-chamber heart model with spatially varying robin boundary conditions to model the effect of the pericardium. J Biomechd 101:109645. 10.1016/j.jbiomech.2020.109645 10.1016/j.jbiomech.2020.109645 PMC7677892 32014305

[CR85] Tanaka T, Arakawa M, Suzuki T et al (1986) Compliance of human pulmonary “venous” system estimated from pulmonary artery wedge pressure tracings. Comparison with pulmonary arterial compliance. Jpn Circ J 50(2):127–139. 10.1253/jcj.50.127 3088296 10.1253/jcj.50.127

[CR86] Torbati S, Daneshmehr A, Pouraliakbar H et al (2024) Personalized evaluation of the passive myocardium in ischemic cardiomyopathy via computational modeling using Bayesian optimization. Biomech Model Mechanobiol 23(5):1591–1606. 10.1007/s10237-024-01856-0 38954283 10.1007/s10237-024-01856-0

[CR87] Vinnakota KC, Bassingthwaighte JB (2004) Myocardial density and composition: a basis for calculating intracellular metabolite concentrations. Am J Physiol-Heart Circulatory Physiol 286(5):H1742–H1749. 10.1152/ajpheart.00478.2003 10.1152/ajpheart.00478.2003 14693681

[CR88] Vitello DJ, Ripper RM, Fettiplace MR et al (2015) Blood density is nearly equal to water density: a validation study of the gravimetric method of measuring intraoperative blood loss. J Vet Med 2015:1–4. 10.1155/2015/152730 10.1155/2015/152730 PMC4590883 26464949

[CR89] Wang JJ, Flewitt JA, Shrive NG et al (2006) Systemic venous circulation. Waves propagating on a windkessel: relation of arterial and venous windkessels to systemic vascular resistance. Am J Physiol-Heart Circulatory Physiol 290(1):154–162. 10.1152/ajpheart.00494.2005 10.1152/ajpheart.00494.2005 16113064

[CR90] Westerhof N, Bosman F, De Vries CJ et al (1969) Analog studies of the human systemic arterial tree. J Biomech 2(2):121–143. 10.1016/0021-9290(69)90024-4 16335097 10.1016/0021-9290(69)90024-4

[CR91] Westerhof N, Lankhaar JW, Westerhof BE (2009) The arterial Windkessel. Med Biol Eng Comput 47(2):131–141. 10.1007/s11517-008-0359-2 18543011 10.1007/s11517-008-0359-2

[CR92] Wetterer E (1940) Quantitative Beziehungen zwischen Stromstärke und Druck im natürlichen Kreislauf bei zeitlich variabler Elastizität des arteriellen Windkessels; Wetterer, Erik. JF Lehmanns Verlag, Dr. med

[CR93] Wymer DT, Patel KP, Burke WF et al (2020) Phase-contrast MRI: physics, techniques, and clinical applications. Radiographics 40(1):122–140. 10.1148/rg.2020190039 31917664 10.1148/rg.2020190039

[CR94] Xi J, Lamata P, Niederer S et al (2013) The estimation of patient-specific cardiac diastolic functions from clinical measurements. Med Image Anal 17(2):133–146. 10.1016/j.media.2012.08.001 23153619 10.1016/j.media.2012.08.001 PMC6768802

[CR95] Young DB (2010) Control of cardiac output. Colloquium Ser Integr Syst Physiol From Mole Funct 2(1):1–97. 10.4199/C00008ED1V01Y201002ISP006

[CR96] Zerhouni EA, Parish DM, Rogers WJ et al (1988) Human heart: tagging with MR imaging-a method for noninvasive assessment of myocardial motion. Radiology 169(1):59–63. 10.1148/radiology.169.1.3420283 3420283 10.1148/radiology.169.1.3420283

[CR97] Zhang Y, Wang VY, Morgan AE et al (2020) A novel MRI-based finite element modeling method for calculation of myocardial ischemia effect in patients with functional mitral regurgitation. Front Physiol 11:158. 10.3389/fphys.2020.00158 32231584 10.3389/fphys.2020.00158 PMC7082816

[CR98] Zingaro A, Ahmad Z, Kholmovski E et al (2024) A comprehensive stroke risk assessment by combining atrial computational fluid dynamics simulations and functional patient data. Sci Rep 14(1):9515. 10.1038/s41598-024-59997-2 38664464 10.1038/s41598-024-59997-2 PMC11045804

